# Modulation of nitric oxide synthase activity in macrophages

**DOI:** 10.1155/S0962935195000135

**Published:** 1995-03

**Authors:** P. G. Jorens, K. E. Matthys, H. Bult

**Affiliations:** 1 Division of Pharmacology Department of Medicine University of Antwerp (UIA) Wilrijk B-2610 Belgium; 2 Department of Intensive Care Medicine University Hospital of Antwerp Belgium

## Abstract

L-Arginine is converted to the highly reactive and unstable nitric oxide (NO) and L-citrulline by an enzyme named nitric oxide synthase (NOS). NO decomposes into other nitrogen oxides such as nitrite (NO_2_^-^) and nitrate (NO_2_^-^), and in the presence of superoxide anion to the potent oxidizing agent peroxynitrite (ONOO^−^). Activated rodent macrophages are capable of expressing an inducible form of this enzyme (iNOS) in response to appropriate stimuli, i.e., lipopolysaccharide (LPS) and interferon-γ (IFNγ). Other cytokines can modulate the induction of NO biosynthesis in macrophages. NO is a major effector molecule of the anti-microbial and cytotoxic activity of rodent macrophages against certain micro-organisms and tumour cells, respectively. The NO synthesizing pathway has been demonstrated in human monocytes and other cells, but its role in host defence seems to be accessory. A delicate functional balance between microbial stimuli, host-derived cytokines and hormones in the microenvironment regulates iNOS expression. This review will focus mainly on the known and proposed mechanisms of the regulation of iNOS induction, and on agents that can modulate NO release once the active enzyme has been expressed in the macrophage.


Mediators of Inflammation 4, 75–89 (1995)

### Modulation of nitric oxide synthase activity in macrophages

P. G. Jorens,* K. E. Matthys and H. Bult[CA]

Division of Pharmacology, Department of Medicine, University of Antwerp (UIA), B-2610 Wilrijk, Belgium
*Current address: Department of Intensive Care Medicine, University Hospital of Antwerp, Belgium

[CA] Corresponding Author

**L-Arginine is converted to the highly reactive and unstable nitric oxide (NO) and L-citrulline by an enzyme named nitric oxide synthase (NOS). NO decomposes into other nitrogen oxides such as nitrite ($NO_2^-$) and nitrate ($NO_3^-$), and in the presence of superoxide anion to the potent oxidizing agent peroxynitrite ($ONOO^-$). Activated rodent macrophages are capable of expressing an inducible form of this enzyme (iNOS) in response to appropriate stimuli, i.e., lipopolysaccharide (LPS) and interferon-γ (IFNγ). Other cytokines can modulate the induction of NO biosynthesis in macrophages. NO is a major effector molecule of the anti-microbial and cytotoxic activity of rodent macrophages against certain micro-organisms and tumour cells, respectively. The NO synthesizing pathway has been demonstrated in human monocytes and other cells, but its role in host defence seems to be accessory. A delicate functional balance between microbial stimuli, host-derived cytokines and hormones in the microenvironment regulates iNOS expression. This review will focus mainly on the known and proposed mechanisms of the regulation of iNOS induction, and on agents that can modulate NO release once the active enzyme has been expressed in the macrophage.**

### History and basic concepts

The history of the discovery of the L-arginine–NO pathway is discussed only briefly, as excellent reviews are available.[1–3] In the early 1980s, *in vivo* studies demonstrated that rats, mice and humans excrete more nitrate than they ingest, suggesting that mammals form nitrogen oxides endogenously.[1,3] In 1987, NO was found to explain both the biologica activity of the elusive endothelium-derived relaxin factor[2–5] and the L-arginine dependent tumouricida activity of activated murine macrophages.[6–8] Th products of this enzymatic pathway are L-citrullin and the highly reactive and unstable nitric oxid (NO), which decomposes (after complexing wit certain forms of iron, or non-enzymatically) int other nitrogen oxides such as nitrite ($NO_2^-$) an nitrate ($NO_3^-$) (Fig. 1). The enzyme responsible fo NO production has been named nitric oxide synthas (NOS).[2] Although evidence exists that NO is th primary product released by NOS, it is possible tha NOS-containing cells produce a mixture of NO, NO, $NO_2^-$, $NO_3^-$, $N_2O_3$, nitrosamines, non-protei nitrosothiols and *S*-nitrosylated proteins.[3]

Soon after its discovery, the new metabolic pathway was found to be L-arginine dependent.[5,9–13] NO is synthesized from the terminal guanidino nitrogen atom of this amino acid (and not D-arginine),[2] without loss of the guanidino carbon atom.[6] Although it was initially believed that cNOS produced NO by a different process without nucleotide-derived cofactors, recent work now supports a general scheme.[12] NOS incorporates molecular oxygen both into NO and citrulline. Activated macrophages form NO from ω-hydroxy-L-arginine,[12] confirming the proposal that this compound is an intermediate in the biosynthesis of NO.[10]

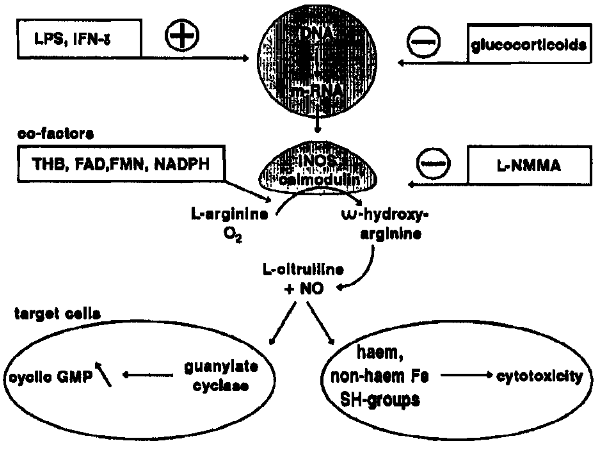

FIG. 1. Schematic representation of the induction of the nitric oxide mediated effector pathway in rodent macrophages, and its inhibition by glucocorticoids and arginine analogues, such as L-monomethyl-arginine (L-NMMA). Interaction of NO with the haem group of cytoplasmic guanylate cyclase stimulates cyclic GMP formation, while its interaction with other haem groups, or with non-haem bound iron (Fe) can lead to cytotoxic effects. THB, tetrahydrobiopterin.

© 1995 Rapid Communications of Oxford Ltd

Soon it became clear that this metabolic pathway exists in various cells from different embryological origins, and that different forms of NOS can be distinguished, based on their expression and mode of NO release.[2,3] A first subclass consists of $Ca^{2+}$- and calmodulin dependent enzymes (cNOS), releasing NO within seconds when calmodulin binds to the enzyme in response to a rise of cytoplasmic $Ca^{2+}$ levels upon receptor stimulation. These forms are constitutively expressed in endothelial cells and certain neuronal cells. The NO produced by these cells acts as an inhibitory signal: it stimulates a cytoplasmic guanylate cyclase, and cyclic GMP is formed as second messenger.[2] The second subclass (iNOS) consists of inducible, $Ca^{2+}$-independent enzymes, which can be expressed in most nucleated cells, including rodent macrophages, following induction with cytokines.[2,14] L-Homoarginine can serve as a substrate for iNOS, but not for cNOS.[2,15]

Recently, NOS has been purified and cloned[16,22] from cerebellum, endothelium, rodent macrophages and human hepatocytes (reviewed by Nathan[3]). NOS has a close homology to cytochrome $P_{450}$ reductase.[17] The biochemical and molecular biological reports on NOS result in a confusing synopsis with respect to a scale of variables: specific activity, $K_m$ for L-arginine, relative dependence on $Ca^{2+}$ and co-factors, subcellular distribution or the gene regulating the expression of this enzyme. Until the cloning from additional cells and species and the use of antibody probes clarify the systematics of the various forms of NOS, the division into two subclasses (cNOS and iNOS) can be retained as the most useful one.[3]

The murine iNOS macrophage enzyme displays approximately 50% sequence homology to the cNOS of neuronal origin.[19] Macrophage iNOS mRNA is strikingly inducible and it is absent in quiescent macrophages. Like neuronal NOS, macrophage NOS has recognition sites for FAD, FMN and NADPH and has a consensus calmodulin binding site.[18–22] Calmodulin binds tightly as a subunit to iNOS, which explains why iNOS is $Ca^{2+}$ independent, in contrast to cNOS.[23]

Macrophages from the cloned macrophage cell line RAW264.7 from Albeson leukaemia virus-induced BALB/c lymphocytic lymphoma are rather exceptional as they also express cNOS activity, which is $Ca^{2+}$ dependent and decreases after activation with IFNγ or LPS, or with increasing passage number.[24]

Many studies have shown that iNOS expression in macrophages is tightly regulated by a delicate balance between microbial stimuli, host-derived cytokines and hormones in the micro-environment. This review will therefore focus on the known and proposed mechanisms of the regulation of iNOS expression in macrophages and on the agents that can modulate NO release in these cells.

### Functions of nitric oxide in host defence

Nitric oxide is a major effector molecule of the antimicrobial and cytotoxic activity of rodent macrophages against intracellularly growing microbes, some extracellular parasites,[25,26] viruses[27,28] and some tumours.[1] Haem proteins, proteins containing non-haem Fe and DNA are the molecular targets of NO in cells in general and in tumour cells in particular.[1,3] These interactions have many consequences in the target cell: decreased protein synthesis,[29,30] inhibition of ribonucleotide reductase and hence DNA synthesis,[1] suppression of mitochondrial respiration and aconitase of the citric acid cycle,[1,3,25] modulation of the post-transcriptional regulation of genes involved in iron homeostasis,[31] activation of RNA binding by an iron regulatory factor,[32,33] the suppression of antibody synthesis[34] and inhibition of T-cell proliferation.[35] NO can also combine with superoxide anion to form the potent oxidizing agent peroxynitrite ($ONOO^-$).[36] It is interesting to observe that macrophages, the prototypical effector cells for NO-mediated cytotoxicity, are themselves targets for NO or peroxynitrite, as they die prematurely in culture when activated to express iNOS, a process that appears to be mediated through apoptosis.[37,38] Moreover, NOS activity in macrophages is associated with glucose depletion, glycolysis and hexose monophosphate shunt activity, a decreased flux of glucose through the tricarboxylic acid cycle and the induction of glucose-6-phosphate dehydrogenase.[39,40] These results demonstrate that energy metabolism in macrophages can, to a significant extent, be determined by products of iNOS. A regulatory loop exists between iron metabolism and NO in macrophages, as the addition of iron to macrophages incubated with LPS/IFNγ significantly reduces their ability to produce NO.[41]

Induction of NOS in monocytes or tissue macrophages *in vivo* has been demonstrated or suggested by indirect measurements in several animal models. These include intravenous injection of LPS,[42,43] oral endrin administration to rats,[44] IgA immune-complex vasculitis of the lung,[45] tracheal bleomycin instillation,[46] diabetes,[47] treatment with cytotoxic drugs,[48,49] exposure to benzene[50] or inhalation of ozone.[51] The role of endogenous NO in inflammation is still unclear. An inhibitor of NOS activity enhanced neutrophil adhesion to endothelial cells and their subsequent extravasation by upregulation of the expression of the CD11/CD18 integrin on the neutrophil,[52] suggesting that endogenous NO continuously suppresses neutrophil adhesion. In contrast, the same inhibitor blocked neutrophil-dependent injury of the pulmonary vasculature[45] and the oedema induced by intradermal injection of substance P.[53] The pro- and anti-inflammatory activity of endogenous NO deserves further investigation in different experimental settings.

### Atherosclerosis and iNOS

Oxidatively modified low-density lipoprotein (oxLDL) is taken up by macrophages via unregulated receptor-mediated endocytosis.[54] The lipid-laden macrophages are transformed to foam cells, which are present in early atherosclerotic lesions. Macrophages are capable of accelerating LDL oxidation *in vitro*. NO, particularly when generated in the presence of superoxide anion, can oxidize LDL as well, since the highly reactive peroxynitrite is generated.[36] However, NO synthesis by macrophages is not required for macrophage-mediated oxidation of LDL *in vitro*. On the contrary, it seems to exert a protective role in preventing oxidative LDL modification by macrophages.[55–57] It has been proposed that NO can inhibit LDL oxidation by acting as a chain-breaking anti-oxidant that is capable of scavenging carbon-centred and peroxyl radicals.[58]

Moreover, uptake of oxLDL, but not the acetylated form, by macrophages has been shown to modulate iNOS activity. OxLDL may prime the macrophage for enhanced, presumably prostaglandin-mediated NO biosynthesis, as assessed by nitrite and citrulline accumulation in the supernatant.[59] On the other hand, it suppresses iNOS activity resulting from stimulation with LPS or IFNγ.[60–62] Addition of the glucocorticoid receptor antagonist mifepristone did not prevent the oxLDL dependent iNOS inhibition, indicating that the glucocorticoid receptor is not involved in the suppressive effect of oxLDL.[63] Failure to detect NO production by those macrophages appears to result from lack of NO synthase activity.[64]

Recent evidence suggests that iNOS activity, which is normally not present in the arterial wall, is expressed when macrophage-rich lesions develop. The cholesterol-induced formation of foam cell-rich fatty streaks in the rabbit aorta leads to the induction of a non-endothelial NOS.[65] Furthermore, balloon angioplasty and denudation of, respectively, rabbit[66] and rat[67] carotid artery, which induces intimal thickening with a prominent influx of macrophages, is accompanied by induction of NOS activity. Yet this does not prove that macrophages are the source of NO, since iNOS can be induced in smooth muscle cells as well. The continuous NO release leads to hyporeactive contractile responses, but its consequences for the development of atherosclerotic lesions remain to be determined.

### Induction of iNOS in rodent macrophages

*Direct inducers:* A first, critical level of regulation consists of the induction process, since iNOS is normally not expressed in resting macrophages. LPS, the major constituent of the outer wall of Gram-negative bacteria[68] and IFNγ[7] are potent inducers of iNOS, and therefore often employed to elicit NO biosynthesis in macrophages. Contamination with the omnipresent LPS can thus be a serious and complicated technical problem when studying the regulation of iNOS activity. Other bacterial products, such as exotoxins of Gram-positive bacteria, toxic shock syndrome toxin-1, enterotoxin B and lipoteichoic acid,[69–71] high molecular weight material derived from *Mycoplasma fermentans*[72] and synthetic analogues of the N-terminal part of bacterial lipoprotein[73] are direct inducers of iNOS in macrophages as well. A few cytokines, such as IFNγ[7,74] and migration inhibitory factor (MIF)[75] can directly activate macrophages to synthesize NO. Other inducers include $Ca^{2+}$ ionophore, which both induces and enhances the potency of LPS to induce $NO_2^-$ production,[76] certain antitumour agents such as flavone-8-acetic acid,[77] certain food proteins,[78,79] asbestos fibres,[80] stable analogues of cyclic AMP[81] (see subsequent section), monoclonal antibodies to MHC II antigens[82] and membrane fragments of tumour cells.[83]

In primary mouse peritoneal macrophages, LPS and IFNγ only caused significant biosynthesis of nitrogen oxides when the cells were elicited by thioglycolate broth or pretreated with *Bacillus Calmette–Guérin*.[8] Based on these experiments with mouse cells, it appears that macrophage activation for cytotoxicity requires two exogenous signals: one signal primes or sensitizes the cell, the second activates it for killing and production of NO.[1,8] In rats, however, resident alveolar and peritoneal macrophages produce NO in response to relatively low doses of a single exogenous activating stimulus, including LPS.[84,85] Moreover, the macrophage activation process may differ between mouse strains,[86] and appears to be age-dependent. The ability of cultured macrophages to secrete nitrogen oxides correlates with the age of the mice from which they are harvested. IFNγ-induced release of NO is 50% lower in macrophages taken from old mice than from young mice.[87]

*Signal transduction:* The induction of NOS in macrophages requires protein synthesis.[2,8,9,11] When added during the induction phase, inhibitors of ADP ribosylation are also able to inhibit nitrite production.[88,89] Certain protein synthesis inhibitors can trigger an immediate early gene response and efficiently activate transcription of iNOS.[90] The mRNA for iNOS shares some features with mRNA of cytokines such as the transient expression and decay of its mRNA (half-life of approximately 6 h) which can be prevented by protein synthesis inhibition.[91] The signal transduction pathway by which IFNγ induces iNOS expression remains to be established. Experimental evidence suggests the involvement of G-proteins and phospholipase C. Experiments with the $G_i$-protein inhibitor pertussis toxin suggest that LPS stimulates tumour necrosis factor-α (TNFα) and NO production in mouse peritoneal macrophages through different biochemical pathways, but the signal transduction for both pathways is regulated by a pertussis toxin-sensitive factor,[92,93] presumably a $G_i$-protein. NO biosynthesis is dose-dependently reduced by protein kinase C inhibition and induced by phorbol esters, activators of protein kinase C,[94] as well as by translocation of protein kinase C via activation of the membrane protein CD53, providing evidence that protein kinase C is involved in iNOS induction.[95,96] Analysis of diacylglycerol synthesis provided direct evidence that NO synthesis in macrophages involves the activation of an unusual phosphatidylcholine-specific phospholipase C.[97] However, tyrosine phosphorylation may also participate in the induction of NOS, as protein tyrosine kinase inhibitors (of the tyrophostin AG 126 family) protect mice against LPS-induced lethal toxicity, correlating with the ability of these agents to block LPS-induced NO production in macrophages.[98,99] Macrophages from mice with a targeted disruption of the IFN regulatory factor 1-gene produced very little NO.[100] The promotor of the murine iNOS gene contains an NF-kappa B site, designated NF-kappa Bd. Pyrrolidine dithiocarbamate, an inhibitor of NF-kappa B, blocks both the activation of NF-kappa Bd-binding proteins and NO biosynthesis in LPS-treated macrophages.[101,102]

*Interactions between stimuli:* Bacterial LPS interacts synergistically with IFNγ to induce NOS when both stimuli are added together.[8,74,103] A recombinant $NH_2$-terminal fragment of bactericidal/permeability-increasing protein, which binds to LPS in the outer membrane of Gram-negative bacteria, was shown to inhibit murine macrophage nitric oxide production elicited by LPS plus IFNγ,[104] while the rabbit plasma LPS-binding protein, which enhances binding and functional responses to LPS, enhanced this NO production.[105] The elevated expression of iNOS mRNA after co-stimulation has been shown to be due to increased stability by some investigators, but due to a much higher rate of transcription of the NOS gene by others.[41,106] This synergism of LPS can be mimicked by a monoclonal antibody directed against the 73 kDa LPS receptor on murine leukocytes,[107] which identifies this 73 kDa protein as a receptor that mediates LPS-induced changes in macrophage NO production. IFNγ has been identified as the major priming factor when IFNγ, LPS and TNFα are added together to the macrophage.[108]

Another regulatory mechanism is the observation that pretreatment of macrophages with low doses of LPS can selectively reprogram these cells, i.e., down-regulate for subsequent LPS-activated NO production,[92] a mechanism refractory to pertussis toxin. Pre-exposure of peritoneal macrophages to low concentrations of LPS also suppresses the subsequent induction of iNOS by IFNγ. These findings suggest that preactivation of pathways normally contributing to synergistic induction of NOS may deplete macrophages of factors needed for its expression.[109] Regulation of NOS *in vivo* may therefore depend on the relative tempo with which the inflammatory and immune responses evolve. Reduced NO production by macrophages exposed to LPS has also been shown to markedly reduce the ability of macrophages to kill the intracellular parasite *Leishmania major*. This endotoxin tolerance may represent an important means of regulation of NO synthesis and thus a survival mechanism for intracellular parasites.[110]

Phagocytosis itself may cause upregulation of iNOS induction. Phagocytosis of *Leishmania enrietti* promastigotes or latex beads by murine macrophages enhanced IFNγ-stimulated nitrite production and could be an important mechanism of up-regulating their microbicidal activity.[111] Ingestion of zymosan, but not of latex beads or silica, also acted synergistically with LPS to induce iNOS activity.[112] These results demonstrate that phagocytosis, although capable, is not always sufficient to provide an additional signal for the induction of iNOS in mouse macrophages. In rat macrophages opsonized zymosan induced nitrite production in the absence of further stimuli.[85]

*Autocrine and paracrine regulation by TNF:* Conflicting results have been reported on the ability of TNFα to induce NOS in murine macrophages. Some authors reported substantial amounts of nitrite in supernatants of mouse macrophages stimulated with this cytokine alone.[113] Others did not find TNFα or TNFβ to induce NO production by itself, but TNF is believed to serve as an autocrine signal for cytokine induced NO production in murine macrophages. Endogenously produced TNF is also involved in the induction of NO effector mechanisms when muramyl dipeptide (MDP),[103] a bacterial wall constituent, or *Leishmania* promastigotes[111] are added as co-stimuli with IFNγ. For *Leishmania* and *Toxoplasma*, the parasite itself participates in the regulation of NO production by macrophages through autocrine TNFα induction by the parasite: NO synthesis by IFNγ treated cells can be blocked by monoclonal antibodies to TNFα[114–116] and TNFα potentiates the ability of IFNγ to reduce the development of cutaneous Leishmaniasis *in vivo* via an L-arginine dependent pathway.[117,118] These results are in agreement with the results obtained with lymphokines in the activation of macrophages against trypanosomes: T-cell derived TNFα and IFNγ synergistically activate mouse macrophages for the killing of intracellular

*Trypanosoma cruzi* through an NO-mediated mechanism.[119] The complement subcomponent C1q may be involved in the modulation of autocrine binding of TNF for subsequent generation of cytotoxic NO: pretreatment of macrophages with 3,4,-dehydro-D,L-proline, an inhibitor of C1q secretion, suppresses lipid A-induced activation for cytotoxicity which correlates with a decreased NO production and reduction in their capacity to bind TNF.[120] The role of TNF in macrophage-mediated cytotoxicity is thus not limited to its lytic action on certain target cells, but TNF also acts with IFNγ as an autocrine immunomodulator to elicit the conversion of L-arginine into nitrogen oxides.[121]

In addition, paracrine effects of TNF have been documented. TNFα or TNFβ fail to induce NO production by themselves, but addition of either cytokine to IFNγ increased nitrite production compared with IFNγ alone.[74,122] In contrast to the paracrine effects of TNF in murine macrophages, TNF exerts no or only a small enhancing effect on IFNγ-induced NO production by resident rat macrophages.[84,123]

*Synergy with* α *and* β *interferons:* IFNα or IFNβ, in combination with LPS, also raised LPS-induced nitrite production.[74] A monoclonal antibody specific for IFNβ inhibited LPS-induced NO production in thioglycolate-elicited peritoneal macrophages, which supports the concept that IFNβ also provides an essential signal for LPS-triggered NO production by mouse macrophages.[124] However, neither IFNβ nor TNFα, alone or in combination, triggered NO production, demonstrating again that these macrophage-derived cytokines, while necessary, are by themselves not sufficient to induce iNOS in these murine cells.

*Other stimulating cytokines:* Granulocyte–macrophage colony stimulating factor (GM–CSF)-elicited, bone marrow-derived mouse macrophages required only LPS for effective killing of K562 cells, but produced little nitrite in response to LPS, unless treated with IFNγ. Conversely, macrophage colony stimulating factor CSF-1-elicited bone marrow macrophages required the classical IFNγ plus LPS treatment protocol to become tumouricidal, but secreted nitrite in response to high concentrations of LPS alone.[125,126] Moreover, GM–CSF, but not CSF-1-derived macrophages, showed an L-arginine dependent Listeriacidal activity. Macrophage colony stimulating factor- and GM–CSF-derived bone marrow macrophages treated with cisplatin are effective in the production of NO and the generation of tumouricidal activity.[127] GM–CSF is also an efficient enhancer and primer of iNOS activity in rat alveolar macrophages.[123]

When interleukin-2 (IL-2) was combined with IFNγ and TNFα, there was a marked cooperative induction of mRNA and iNOS enzyme activity in murine peritoneal macrophages. This cooperation was truly synergistic, as the full combination was many times more effective than the individual agents or paired combinations. As it required protein synthesis, the intermediate expression of new gene products is suggested.[128] Indeed, endogenous production of TNFα was required for this cooperative effect of IL-2 on IFNγ-induced NO production and tumour cell lysis.[129] Moreover, certain susceptible tumour targets constitutively produce one or more soluble recognition factors that synergize with the natural cytokines IFNγ and IL-2 to render macrophages cytotoxic for the target cell.[130]

A recombinant form of human MIF, a lymphokine produced by antigen-stimulated lymphocytes which suppresses macrophage migration *in vitro*, induced murine macrophages to express iNOS and to produce high levels of NO.[75] However, another group reported that MIF-mediated migration inhibition was not accompanied by endogenous production of NO or TNF.[131] Inhibition of murine macrophage migration was also triggered by lipid A, the lipid moiety of LPS, after priming with IFNγ, whereas neither MIF nor IFNγ inhibited migration when given alone.[131] Priming with IFNγ induced the biosynthesis of NO and TNF. This resembled the effects of IFNγ on the induction of NO-dependent tumour cytotoxicity in mouse macrophages, which requires autocrine stimulation by TNF as well.

*Inhibitory cytokines:* IL-4, IL-10, IL-13, transforming growth factor-β (TGFβ) and macrophage deactivating factor have been shown to inhibit the induction of NO production in macrophages.[132–137] The effects of these cytokines on macrophage function are more complex than previously recognised. IL-4 is able to inhibit NO synthesis by IFNγ-stimulated murine peritoneal[138] and adherent splenic[134] macrophages, while evidence has also been presented for synergism between IL-4 and IFNγ for the induction of the L-arginine-dependent killing of *Leishmania major* amastigotes.[139] The repression of iNOS induction and NO biosynthesis by IL-10 is effective only when cells are pretreated with this cytokine[135] and the mechanism of this action was identified as inhibition of endogenous TNFα production.[140] IL-10 also has, however, the capacity to enhance the early production of NO by macrophages co-stimulated with IFNγ and exogenous TNFα.[141] These data support the notion that the cytokines IL-4 and IL-10, secreted by the $T_h2$ lymphocyte subset, might modulate NO synthesis induced by IFNγ, an effector of $T_h1$ cells.[142] Moreover, the combination of sub-optimal concentrations of any two of the cytokines IL-4, IL-10 and TGFβ gave a potent synergistic effect on the suppression of NO production and killing of schistosomes by IFNγ-treated macrophages.[143]

TGFβ inhibited NO synthesis in IFNγ-activated murine macrophages, but only at high concentrations (100 ng/ml),[132,144,145] while it enhanced iNOS induction in 3T3 fibroblastic cells.[146] On the other hand induction of iNOS by MIF is highly sensitive to the inhibitory effect of TGFβ. This observation is a further indication that macrophage activation by IFNγ and MIF occurs through different signalling pathways.[75] TGFβ has been demonstrated to reduce NOS specific activity and protein in both cytosolic and particulate fractions, to reduce iNOS mRNA and translation by decreasing the stability of mRNA, and by degrading the NOS protein.[147]

Monocyte chemotactic protein 1, a potent chemoattractant for monocytes, is able to inhibit the production induced by LPS and IFNγ in a dose-dependent manner, but this effect is only achieved when the cells are pretreated with the chemoattractant.[148]

*Up-regulation by miscellaneous agents:* Elevated temperature may contribute to enhanced host defence by accelerating and amplifying the induction of NO synthesis in macrophages.[149] Picolinic acid, a catabolite of L-tryptophan, while being ineffective by itself, augmented IFNγ-induced nitrite production via both TNFα-dependent and -independent mechanisms. This provides further evidence for possible connections between the tryptophan and arginine dependent cytotoxic effector pathways in murine macrophages.[150] Binding of extracellular nucleotides, including ATP, to purinergic receptors may increase NO production by macrophages.[151] This effect might occur in pathological conditions where significant amounts of ATP can be released due to cellular damage.

An L-arginine terminal synthetic compound belonging to the family of hypoxanthine derivatives enhanced IFNγ-induced nitrite release from cultured peritoneal macrophages.[152] Muramyldipeptide (MDP), a constituent of bacterial cell walls has been shown to enhance both LPS[153] and IFNγ-induced[123] NO production. A cytotoxic substance, isolated from *Bacillus stearothermophilus* and identified as bis(2-hydroxyethyl)trisulphide, increased NO formation by macrophages.[154] The macrophage activating tetrapeptide tuftsin has been shown to synergize with IFNγ for NO production in murine, but not in rat macrophages.[123,155]

Ligated complement receptor type 3 (CR3) and IFNγ act synergistically to induce NO production and CR3 mediates the group B streptococcus-induced signal for NO production in IFNγ-treated macrophages.[156] Bafilomycin A1 (BAF), an inhibitor of vacuolar-type-H(+)-ATPases, causes an increase in intravesicular pH and enhances nitrite release by activated macrophages. However, the NO concentration necessary to kill parasites was higher in BAF-exposed than in control macrophages, suggesting that microbicidal nitrogen derivatives were less active at alkaline pH.[157] The anti-cancer drug taxol, which blocks cell division by stabilizing microtubules, synergized with INFγ, like LPS, to activate macrophages for L-arginine-dependent tumour lysis.[158] Conversely, nocodazole and colchicine, two chemically distinct microtubule depolymerizing agents, completely prevented LPS-induced NO production in vascular smooth muscle cells.[159]

*Regulation by prostanoids and other lipids:* The hypothesis that platelet activating factor (PAF) acts as auto- or paracrine up-regulator of iNOS, has been substantiated by the finding that WEB 2086, an antagonist of PAF receptors, attenuated LPS-stimulated NO biosynthesis in cultured murine macrophages.[160] Thus, inhibition of NO induction may contribute to the beneficial effects of PAF antagonists in endotoxaemia.

Macrophages derived from mice fed with a diet rich in n-3 polyunsaturated fatty acids produce more $NO_2^-$ in response to IFNγ compared with macrophages from the control group.[161] Bronchoalveolar macrophages from rats fed diets rich in n-3 fatty acids produce significantly more NO than macrophages isolated from n-6 polyunsaturated fatty acids fed animals: changes in n-6-derived prostanoids may account for these data.[162]

However, the regulation of iNOS activity by arachidonic acid metabolites is still controversial. Nitrite production by peritoneal mouse macrophages was suppressed by phospholipase $A_2$ inhibitors, 5-lipoxygenase inhibitors and a glutathione-*S*-transferase inhibitor, while inhibitors of 12- and 15-lipoxygenase, and of cyclooxygenase were without effect.[163,164] However, Gaillard *et al.* reported that $PGE_2$ and dibutyryl-cyclic AMP, a stable analogue of cyclic AMP, enhanced LPS-induced $NO_2^-$ secretion by rat Kupffer cells.[165] The stimulation is maximal when $PGE_2$ is added after LPS. Cyclic AMP appeared to be a positive signal for iNOS expression in rat vascular smooth muscle, as well.[166,167] Others, however, have reported that $PGE_2$, the stable prostacyclin analogue iloprost, the cyclic AMP mimetic 8-bromo-cyclic AMP and the non-selective phosphodiestrease inhibitor iso-butylmethylxanthine (IBMX) inhibited the LPS-stimulated induction of NOS in murine macrophages. $PGF_{2\alpha}$, a stable analogue of thromboxane $A_2$ and the leukotrienes $B_4$ and $C_4$ were without effect.[168–170]

The hypothesis that cyclic AMP was the second messenger in the down-regulation of NOS by prostaglandins was reinforced by the use of drugs which elevate the intracellular levels of cAMP (8-bromo-cyclic AMP and IBMX). When added during the LPS activation step, these drugs decreased NO

synthase activity.[169] The combination of $PGE_2$ and a phosphodiesterase inhibitor, which induced a prolonged elevation of intracellular cyclic AMP levels, also caused a marked reduction of the NO production by macrophages.[171] The finding that norepinephrine caused a dose-dependent, adrenoceptor-mediated inhibition of the LPS-induced iNOS activity in rat astrocyte cultures, but not in the RAW 264.7 macrophage cell line,[172] is a further indication that cyclic AMP may cause down-regulation of iNOS in some, but not in all cell types.

NO also enhanced cyclooxygenase enzymatic activity in macrophages. This suggests that in conditions in which both NOS and cyclooxygenase become active, there is an NO-mediated increase of the production of pro-inflammatory prostaglandins that may exacerbate inflammatory responses.[173] In respect of this finding, and of the conflicting data on iNOS regulation by prostaglandins, the relationships between eicosanoids and NOS deserve further investigation.

### Regulation of iNOS activity

Once iNOS has been expressed in macrophages, its activity is regulated by several factors, including auto-inactivation, and availability of substrate and cofactors.

*Auto-inactivation:* NO may function as a negative feedback modulator of iNOS activity by interacting with the enzyme-bound haem of iNOS, which may represent a mechanism by which the NOS pathway in activated macrophages is turned off.[174,175] None of 14 monoclonal antibodies raised against iNOS from rat macrophages neutralized iNOS activity, but some of them enhanced enzyme activity through stabilization of the enzyme.[176] Recently, a novel post-translational and non-degradative inactivation of iNOS has been described. LPS, after inducing iNOS, caused macrophages to inactivate the enzyme about 3 days later.[177] The mechanism which remains to be identified is novel as it decreases neither the amount nor the apparent molecular mass of the enzyme.

*Bioavailability of* L*-arginine:* Arginine plays a pivotal role in body protein biosynthesis, biosynthesis of other amino acids and in the urea cycle[1,178] (Fig. 2). Resting macrophages express arginase and the enzyme is up-regulated after immune activation.[179,180] Arginase releases urea from L-arginine (Fig. 2) and might modulate iNOS activity by competition for the substrate in the microenvironment, thereby suppressing macrophage-mediated cytotoxicity.[1] Regenerating wounds have a decreased concentration of L-arginine, elevated ornithine content and high arginase activity, and it has been proposed that the local arginine concentration could have a major effect on cell proliferation rate.[1,178] ω-Hydroxy-L-arginine, the intermediate in the biosynthesis of NO from L-arginine, is a competitive inhibitor of arginase, which may have implications in the regulation of NO levels in cells.[181]

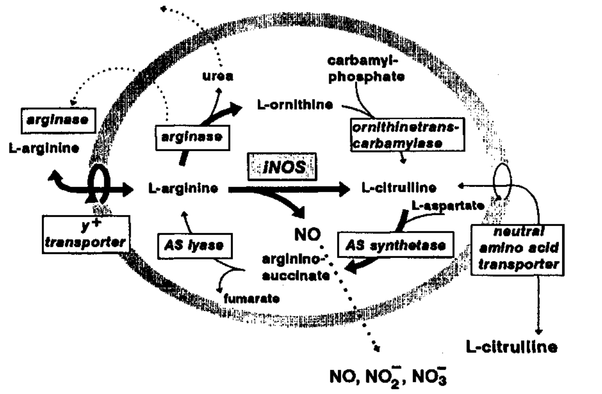

FIG. 2. Pathways of L-arginine metabolism in macrophages. Constitutive enzymes are represented by the open boxes; iNOS is only expressed after immune stimulation of the macrophage. The bold arrows represent enzymes which are up-regulated upon stimulation with IFNγ and/or LPS. AS, arginino succinate.

Citrulline, the by-product of iNOS, can be recycled to arginine by the action of argininosuccinate synthetase and argininosuccinate lyase[178] (Fig. 2). Induction of NOS in macrophages is accompanied by co-induction of activity and mRNA for argininosuccinate synthetase,[182] the rate limiting step in this reaction. The enhanced cellular capacity to regenerate arginine from citrulline could play a significant role in maintaining NO production in macrophages.[111,182,183]

Arginine is transported across cell membranes by system $y^+$, which is $Na^+$-independent and pH-insensitive. The system transports lysine and ornithine as well, and competition for uptake exists among these basic amino acids and L-arginine.[178] Induction of NOS by LPS is accompanied by a marked increase of $y^+$ arginine transporter activity which involves *de novo* synthesis of carrier proteins.[184] This accounts for the accelerated uptake of arginine by murine macrophages from culture medium.[185,186] The factors which control LPS-induced transcription of the arginine transporter gene and the iNOS gene diverge, since only the latter is suppressed by glucocorticoids in activated macrophages.[184] The elevated rate of L-arginine transport into activated cells may provide another mechanism for sustained substrate supply during enhanced utilization of L-arginine.

*Cofactors:* Various inducible and constitutive cytosolic co-factors are required for full iNOS activity. These include the electron donor NADPH, 5,6,7,8-tetrahydrobiopterin (THB), FAD, FMN and glutathione.[11,13,187–189] These findings implicate a redox cycle in which the generation of NO is facilitated.

GTP-cyclohydrolase I, the key step in the biosynthesis of tetrahydrobiopterin, is induced by

IFNγ and LPS in human and rodent macrophages.[190–192] The GTP cyclohydrolase I inhibitor 2,4-diamino-6-hydroxypyrimidine, is capable of inhibiting NO production by macrophages.[189,193] Sepiapterin, a pteridine that does not occur naturally in mammals, but which can substitute tetrahydrobiopterin via a salvage pathway,[194] led to a further increase of NO production even in fully activated macrophages. Phenprocoumon, an inhibitor of sepiapterin reductase, inhibited production of NO by interference with later steps of tetrahydrobiopterin biosynthesis.[189] Because tetrahydrobiopterin is required for the biosynthesis of neurotransmitters, a therapeutic potential of inhibitors of tetrahydrobiopterin for NO-mediated pathological conditions would appear to be limited.[3] Moreover, the addition of high amounts of tetrahydrobiopterin and sepiapterin to activated rat macrophages led to a marked inhibition of NO secretion, which may suggest that iNOS in intact cells is susceptible to feedback regulatory mechanisms.[193]

The flavoprotein inhibitors diphenylene iodonium and its analogues inhibit NO synthesis by mouse macrophages, their lysates and partially purified macrophage iNOS,[195] confirming that iNOS activity depends on an NADPH-utilizing flavoprotein. Proteolytic inhibitors, such as chloromethylketone derivatives, which covalently bind to the active site of serine proteases, can decrease cytotoxicity and nitrite production by both mouse and rat macrophages.[196,197] They have been shown to be effective after induction of the enzyme.[196] As these agents reduce glutathione levels in mononuclear cells[198] the inhibition might be due to depletion of intracellular thiol pools and hence glutathione, one of the co-factors of the iNOS.

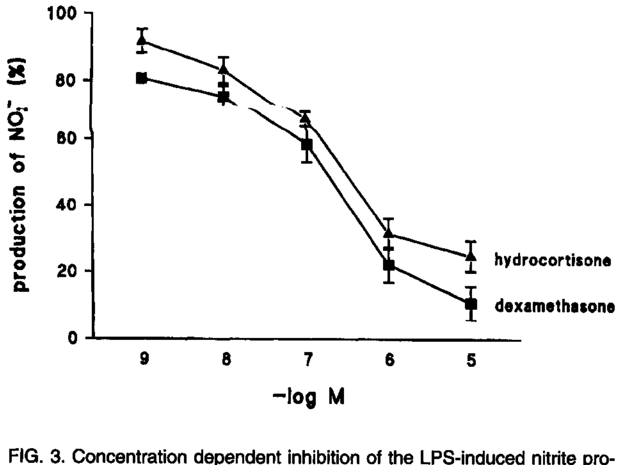

FIG. 3. Concentration dependent inhibition of the LPS-induced nitrite production in rat pulmonary macrophages by either dexamethasone or hydrocortisone. Data from Jorens *et al.*, 1991.[85] ▲, Hydrocortisone; ■, dexamethasone.

### Inhibitors of iNOS induction

*Glucocorticoids:* Corticosteroids are potent suppressors of iNOS induction in macrophages,[184,199,200] (Fig. 3) without influencing the induction of the L-arginine transporter $y^+$.[184] None of the different glucocorticoids used has any effect once the enzyme has been expressed.[85,199] The underlying mechanism is receptor-mediated, since addition of equimolar concentrations of a partial agonist or a full antagonist of the glucocorticoid receptor partially reversed the action of these glucocorticoids.[199,201] At present it is unclear whether transcription of the iNOS gene itself, a transcription factor or a protein which degrades iNOS mRNA is controlled by a glucocorticoid receptor response element. Dexamethasone also blocked IFNγ-induced down-regulation of the mannose receptor expression on macrophages, at least partially, by inhibition of the IFNγ-mediated induction of NO production.[202]

*Miscellaneous inhibitors of iNOS induction:* Co-incubation of murine peritoneal macrophages with mast cell granules during LPS activation dose-dependently inhibited macrophage-mediated tumour cell lysis, and this was associated with a decreased NO production.[203] The inhibitory effect was not due to histamine or serotonin present in the mast cell granules. Cloricromene, a coumarin derivative, which has been found to increase the survival of rats injected with lethal doses of LPS, inhibited the induction but not the activity of the enzyme.[204] Therefore, these agents may have anti-inflammatory/immunosuppressive effects. Pharmacological agents inhibiting iNOS induction in activated macrophages also include the bisbenzylisoquinoline alkaloids, anti-inflammatory constituents of plants of the families Menispermaceae and Ranunculaceae, which have been used as folk remedies in Japan and China.[205] All-trans-retinoic acid, a retinoid with growth-inhibiting and differentiation-inducing properties, inhibited TNF and NO production in peritoneal macrophages.[206]

High concentrations of some dihydropyridine $Ca^{2+}$ channel antagonists, such as nifedipine, as well as a $Ca^{2+}$ channel agonist inhibited the induction of iNOS in phagocytic cells.[207,208] It is unlikely that the effect of nifedipine was dependent on $Ca^{2+}$ channel antagonism, as two other $Ca^{2+}$ antagonists (verapamil and diltiazem) and reduction of extracellular $Ca^{2+}$ with EGTA exerted only a marginal inhibitory effect.[207,208]

### Inhibitors of iNOS activity

*Substrate analogues:* After the discovery of the inhibition of NO formation by $N^G$-monomethyl-L-arginine (L-NMMA),[1,2,209] other L-arginine analogues, i.e., L-$N^G$-nitroarginine (L-NNA), its methyl ester (L-NAME) and $N$-iminoethyl-L-ornithine (L-NIO),[210]

have been shown to inhibit NOS by competition with L-arginine (reviewed by Moncada *et al.*[2] and Nathan[3]). Previous studies have established that iNOS and cNOS may vary in their susceptibility to inhibitory L-arginine analogues.[211] The structure of the $N^G$ substituent appears to determine isoform selectivity.[212–214] L-NNA appeared to be about 300 times less potent as an inhibitor of mouse macrophage iNOS when compared with bovine brain cNOS,[214] whereas L-NMMA is more potent than L-NNA as inhibitor of macrophage iNOS.[211] The irreversible inactivation of iNOS by L-NMMA requires NADPH-dependent hydroxylation.[211,215] Conversely, L-NNA was significantly more potent than L-NMMA as inhibitor of cNOS in vascular endothelial cells. This could be due to the significant metabolism of L-NMMA, but not L-NNA, to L-citrulline in the endothelial cell. L-citrulline is subsequently transformed to L-arginine.[216] This indicates that L-NMMA may serve as a supply of substrate, as well as an inhibitor of NOS under *in vivo* conditions. L-NIO is a potent irreversible inhibitor of both isoforms.[210]

These L-arginine analogues are taken up by macrophages. This is mediated by both the cationic $y^+$ transporter (L-NMMA, L-NIO) and a neutral amino acid transporter (L-NNA, L-NAME).[217,219] L-Arginine transport by system $y^+$ is reduced by L-NMMA and L-NIO, but not affected by L-NNA and L-NAME.[218,219] A neutral amino acid transporter (Fig. 2), with low substrate specificity and insensitive to LPS, mediated the uptake of L-citrulline, L-NNA and L-NAME.[218] Hence, these arginine analogues appear to be not specific for NOS, and may interfere with transport or other aspects of L-arginine metabolism.

The effects of guanidines and uraemic compounds, endogenous NOS inhibitors which may accumulate in renal failure,[220] have been tested on NO production in macrophages. Aminoguanidine, a bifunctional molecule containing the guanido group of L-arginine linked to hydrazine, inhibits iNOS in pancreatic B cells, macrophages, and endothelium-denuded blood vessels of LPS-treated rats and has little effect on cNOS.[221–224] *N,N′*-diaminoguanidine, though being less potent than aminoguanidine as inhibitor of iNOS, appeared to be more selective without effect on cNOS. 1,1-Dimethylguanidine inhibited both iNOS and cNOS, with a potency comparable with L-NMMA.[221] From these data it would appear to be feasible to develop iNOS specific inhibitors, which would be of great help as pharmacological tools for the elucidation of the role of iNOS in pathological conditions.

*Inhibition via cofactors:* Flavoprotein binders, calmodulin binders, haem binders and depletors of tetrahydrobiopterin all have inhibitory activities.[3] Catalase inhibits the activity but not the induction of the enzyme in macrophages stimulated with IFNγ.[225] The inhibition by catalase is reversed in a dose-dependent manner by the addition of the cofactor tetrahydrobiopterin. These results suggest that hydrogen peroxide, which is broken down by the catalase enzyme, may interfere with NO production by affecting the levels of cofactor needed for its synthesis.

*Miscellaneous inhibitors of iNOS:* Some naturally occurring inhibitors include a 5000 MW protein recovered from saliva of the Lyme disease vector *Ixodes dammini*[226] and phosphatidyl serine released by mammary tumour cells.[227] Taurine chloramine, a naturally occurring derivative of taurine, one of the most abundant intracellular free amino acids present in mammalian tissues, inhibits NO secretion and iNOS activity in activated macrophages.[228] This suggests a mechanism through which taurine supplementation may protect against oxidant-induced tissue damage as taurine chloramine is formed by chlorination of taurine by the halide-dependent myeloperoxidase system. Other inhibitors include 3- and 4-amino-1,2,4-triazole, inhibitors of catalase and peroxidases,[229] indazoles,[230] spermine[231] and certain antifungal imidazoles.[232]

*NO as a regulator of macrophage function:* NO may curtail macrophage energy metabolism and viability (*vide supra*), but information on the role of NO as modulator of cytokine production in effector cells is relatively scant. A donor of exogenous NO has been shown to elevate cyclic GMP levels in human macrophages.[233] Correlations between nitrite release and biosynthesis of cytokines by peritoneal macrophages were absent.[234] Physiological levels of NO, as produced by activated mouse macrophages, can selectively down-regulate IL-3 production by spleen cells from contact-sensitized mice, while leaving IL-2 activity unaffected.[235] NO produced endogenously by rat Kupffer cells inhibits the synthesis of IL-6 by these cells in an autocrine way.[236]

NO also inhibits macrophage expression of the major histocompatibility complex class II antigen Ia, which may prevent excessive NO production by inhibiting secretion of IFNγ by T-cells after inhibition of antigen presentation.[237] The ability of anti-Ia antibodies, which may mimic the effect of T-cells, to induce NO production in macrophages suggests that MHC class II molecules act as transmembrane signal transducers finally leading to induction of NOS.[82]

Exposure of primed macrophages to NO-generating compounds results in inhibition of both the consumption of tryptophan and formation of its metabolite kynurenine in the culture medium by inhibiting the haem-containing enzyme indoleamine-2, 3-dioxygenase, the other inducible and amino acid dependent effector pathway with antimicrobial and antitumour activities in macrophages.[238] Thus NO may have an important role as an immunomodulatory as well as effector and autocrine molecule in the immune system.

### Is NO formed by human macrophages?

Reactive nitrogen intermediates are important host defence activities of mouse and rat macrophages, but conclusive proof for NO formation from L-arginine by human macrophages is lacking. Indeed, human monocytes and macrophages acquire cytostatic and anti-microbial activity independent of NO[190,192,239–242] and fail to produce nitrite after stimulation with different cytokines.[243–245] These results suggest that the L-arginine dependent generation of reactive nitrogen intermediates is a species-restricted macrophage mechanism unlikely to participate in the intracellular antimicrobial activity of IFNγ-stimulated human mononuclear phagocytes. Tetrahydrobiopterin biosynthesis is induced in human macrophages[190–192] and deficiency of tetrahydrobiopterin, a cofactor for NOS activity, cannot explain the inability of human macrophages to generate NO. Even exogenous administration of tetrahydrobiopterin failed to elicit NO biosynthesis in human macrophages.[246] Still, two questions remain to be resolved. First, it is unclear whether the Griess reaction, often employed to detect $NO_2^-$ production by macrophages, is sufficiently sensitive. Secondly, it is unclear whether the cytokine profiles used so far to stimulate human macrophages are incomplete and/or distinct from the rodent systems.

Indeed, several lines of indirect evidence suggest the existence of iNOS in human cells.[247] Elevated nitrate levels are found in the plasma and urine of individuals treated with the cytokine IL-2,[248] and in patients suffering from infections or sepsis,[1,249] conditions known to induce IFNγ, TNFα and IL-1 *in vivo*. $CA^{2+}$-independent NOS activity and iNOS mRNA expression have recently been demonstrated in human primary hepatocytes following treatment with LPS and three cytokines,[250] a megakaryocyte cell line[251] and a human colonic epithelial cell line.[252]

Moreover, a number of *in vitro* studies have provided circumstantial evidence for NO generation by human mononuclear cells. Human mononuclear cells inhibit platelet aggregation by releasing a nitric oxide-like factor.[253] TNFα and GM-CSF stimulate human monocytes to restrict growth of *Mycobacterium avium* after incubation for several days, dependent on the generation of nitrite.[254] A virulent strain of *Mycobacterium avium* has been shown to induce NO formation in human monocyte-derived macrophages.[255] Human monocytes are stimulated to release NO upon *in vitro* co-incubation with some tumour cells.[256] Human alveolar macrophages are positive for iNOS in areas of acute and chronic inflammation, as shown by immunohistochemistry of human lung specimens with an antibody directed against cloned murine macrophage iNOS.[257,258] Human mononuclear cells stimulated with glycoprotein 120 of the envelope of human immunodeficiency virus produced small amounts of nitrite.[259] Small quantities of nitrite and citrulline were also generated after incubation of human alveolar macrophages with *Pneumocystis carinii*.[260] Recently, Reiling *et al.*[261] have isolated an iNOS-specific amplification product from human monocytes stimulated with LPS/IFNγ prior to mRNA extraction, as detected by reverse transcriptase polymerase chain reaction. Moreover, analysis of human genomic DNA revealed a specific human iNOS gene.[22,261,262] Finally, IFNγ and TNFα were involved in the activation of the trypanocidal activity of human monocytes through an NO dependent mechanism.[119]

The combined data thus suggest that iNOS activity can be induced to some extent in human macrophages. However, in contrast to the indoleamine-2,3-dioxygenase effector pathway,[190,192,242] the expression of iNOS in human macrophages requires rather specific conditions and limited quantities of NO appear to be generated via the L-arginine dependent effector pathway.

### Conclusion

In the past few years it has become clear that NO, formed from L-arginine by inducible forms of NOS, acts as effector molecule in the cytotoxic and cytostatic activities of rodent macrophages. Since iNOS is normally not constitutively expressed in resting macrophages, transcription and translation of the iNOS gene constitutes the first regulatory level of this pathway. Appropriate stimuli, i.e., microbial constituents and some cytokines, trigger iNOS transcription. Fine tuning of the induction process appears to be complex, and priming, suppression or stimulation of the induction may occur, depending on the cocktail of cytokines present in the macrophage environment. Glucocorticoids inhibit NO release at this level; they repress transcription and/or translation of the iNOS gene. Several mechanisms govern the activity of iNOS once it has become expressed. Auto-inactivation by NO appears to curtail the life-span of active iNOS enzyme. Availability of the substrate L-arginine is another important mechanism of post-translational regulation. Expression of the $y^+$ carrier, which transports basic amino acids across the cytoplasmic membrane, can be up-regulated by the same set of stimuli that induce iNOS transcription. Up-regulation of argininosuccinate synthetase activity, the rate limiting step in the pathway that recycles L-citrulline to L-arginine, is another mechanism to provide iNOS with substrate. Conversely, the same set of stimuli may cause up-regulation of arginase expression and excretion, and remove L-arginine from extracellular and intracellular sources, thereby competing with the $y^+$ carrier and iNOS for the available substrate. It is still unclear to what extent NO influences the biosynthesis of cytokines, prostanoids and other macrophage products, as several reports contradict each other. However, there is little doubt that excessive NO formation will eventually kill the macrophage itself.

Despite intense, often unpublished research, demonstration of the expression of iNOS activity in human macrophages remains troublesome. Inadequate analytical procedures and/or stimuli could form an explanation. Moreover, the role of NO in inflammatory reactions, and its interactions with prostanoids are still controversial. Important progress is, however, to be expected in these fields, once specific pharmacological tools, i.e., substrate analogues with selectively for the inducible isoforms of NOS, or mice in which the iNOS has been knocked out, become available.

### References

1. Hibbs JB Jr, Taintor RR, Vavrin Z, Granger DL, Drapier J-C, Amber IJ, Lancaster JR Jr. Synthesis of nitric oxide from a terminal guanidino nitrogen atom of L-arginine: a molecular mechanism regulating cellular proliferation that targets intracellular iron. In: Moncada S, Higgs EA, eds. *Nitric Oxide from L-Arginine: a Bioregulatory System.* Amsterdam, New York, Oxford: Excerpta Medica, Elsevier Science Publishers, 1990; 189–223.
2. Moncada S, Palmer RMJ, Higgs EA. Nitric oxide: physiology, pathophysiology, and pharmacology. *Pharmacol Rev* 1991; **43**: 109–142.
3. Nathan C. Nitric oxide as a secretory product of mammalian cells. *FASEB J* 1992; **6**: 3051–3064.
4. Ignarro LJ, Byrns RE, Buga GM, Wood KS. Endothelium-derived relaxing factor from pulmonary artery and vein possesses pharmacologic and chemical properties identical to those of nitric oxide radical. *Circ Res* 1987: **61**: 866–879.
5. Palmer RMJ, Ashton DS, Moncada S. Vascular endothelial cells synthesize nitric oxide from L-arginine. *Nature* 1988; **333**: 664–666.
6. Hibbs JB Jr, Taintor RR, Vavrin Z, Rachlin EM. Nitric oxide: a cytotoxic activated macrophage effector molecule. *Biochem Biophys Res Commun* 1988; **157**: 87–94.
7. Stuehr DJ, Gross SS, Sakuma I, Levi R, Nathan CF. Activated murine macrophages secrete a metabolite or arginine with the bioactivity of endothelium-derived relaxing factor and the chemical reactivity of nitric oxide. *J Exp Med* 1989; **169**: 1011–1020.
8. Stuehr DJ, Marletta MA. Induction of nitrite/nitrate synthesis in murine macrophages by BCG infection, lymphokines, or interferon-γ. *J Immunol* 1987; **139**: 518–525.
9. Marletta MA, Yoon PS, Iyengar R, Leaf CD, Wishnok JS. Macrophage oxidation of L-arginine to nitrite and nitrate: nitric oxide is an intermediate. *Biochemistry* 1988; **27**: 8706–8711.
10. Tayeh MA, Marletta MA. Macrophage oxidation of L-arginine to nitric oxide, nitrite, and nitrate. *J Biol Chem* 1989; **264**: 19654–19658
11. Stuehr DJ, Kwon NS, Gross SS, Thiel BA, Levi R, Nathan CF. Synthesis of nitrogen oxides from L-arginine by macrophage cytosol: requirement for inducible and constitutive components. *Biochem Biophys Res Commun* 1989; **161**: 420–426.
12. Leone AM, Palmer RMJ, Knowles RG, Francis PL, Ashton DS, Moncada S. Constitutive and inducible nitric oxide synthases incorporate molecular oxygen into both nitric oxide and citrulline. *J Biol Chem* 1991; **266**: 23790–23795.
13. Stuehr DJ, Kwon NS, Nathan CF. FAD and GSH participate in macrophage synthesis of nitric oxide. *Biochem Biophys Res Commun* 1990; **168**: 558–565.
14. Hauschildt S, Lückhoff A, Mülsch A, Kohler J, Bessler W, Busse R. Induction and activity of NO synthase in bone-marrow-derived macrophages are independent of $Ca^{2+}$. *Biochem J* 1990; **270**: 351–356.
15. Iyengar R, Stuehr DJ, Marletta MA. Macrophage synthesis of nitrite, nitrate and *N*-nitrosamines: precursors and role of the respiratory burst. *Proc Natl Acad Sci USA* 1987; **84**: 6369–6379.
16. Xie Q-W, Cho HJ, Calaycay J, *et al.* Cloning and characterization of inducible nitric oxide synthase from mouse macrophages. *Science* 1992; **256**: 225–228.
17. Bredt DS, Hwant PM, Glatt CE, Lowenstein C, Reed RR, Snyder SH. Cloned and expressed nitric oxide synthase structurally resembles cytochrome P-450 reductase. *Nature* 1991; **351**: 714–718.
18. Hevel JM, White KA, Marletta MA. Purification of the inducible murine macrophage nitric oxide synthase—identification as a flavoprotein. *J Biol Chem* 1991; **266**: 22789–22791.
19. Lowenstein CJ, Glatt CS, Bredt DS, Snyder SH. Cloned and expressed macrophage nitric oxide synthase contrasts with the brain enzyme. *Proc Natl Acad Sci USA* 1992; **89**: 6711–6715.
20. Yui Y, Hattori R, Kosuga K, Eizawa H, Hiki K, Kawai C. Purification of nitric oxide synthase from rat macrophages. *J Biol Chem* 1991; **266**: 12544–12547.
21. Lyons CR, Orloff GJ, Cunningham JM. Molecular cloning and functional expression of an inducible nitric oxide synthase from a murine macrophage cell line. *J Biol Chem* 1992; **267**: 6370–6374.
22. Geller DA, Lowenstein CJ, Shapiro RA, *et al.* Molecular cloning and expression of inducible nitric oxide synthase from human hepatocytes. *Proc Natl Acad Sci USA* 1993; **90**: 3481–3485.
23. Cho HJ, Xie QW, Calaycay J, *et al.* Calmodulin is a subunit of nitric oxide synthase from macrophages. *J Exp Med* 1992; **176**: 599–604.
24. Schmidt HHHW, Warner TD, Nakane M, Förstermann U, Murad F. Regulation and subcellular location of nitrogen oxide synthases in RAW264.7 macrophages. *Mol Pharmacol* 1992; **41**: 615–624.
25. Green SJ, Nacy CA, Meltzer MS. Cytokine-induced synthesis of nitrogen oxides in macrophages: a protective host response to Leishmania and other intracellular pathogens. *J Leukoc Biol* 1991; **50**: 93–103.
26. Liew FY. The role of nitric oxide in parasitic diseases. *Ann Trop Med Parasitol* 1993; **87**: 637–642.
27. Karupiah G, Xie Q-W, Buller RML, Nathan C, Duarte C, MacMicking JD. Inhibition of viral replication by interferon-γ-induced nitric oxide synthase. *Science* 1993; **261**: 1445–1448.
28. Croen KD. Evidence for an antiviral effect of nitric oxide. *J Clin Invest* 1993; **91**: 2446–2452.
29. Billiar TR, Curran RD, Stuehr DJ, Ferrari FK, Simmons RL. Evidence that activation of Kupffer cells results in production of L-arginine metabolites that release cell-associated iron and inhibit hepatocyte protein synthesis. *Surgery* 1989; **106**: 364–372.
30. Billiar TR, Curran RD, Stuehr DJ, West MA, Bentz BG, Simmons RL. An L-arginine-dependent mechanism mediates Kupffer cell inhibition of hepatocyte protein synthesis *in vitro*. *J Exp Med* 1989; **169**: 1467–1472.
31. Drapier J-C, Hirling H, Wietzerbin J, Kaldy P, Kühn LC. Biosynthesis of nitric oxide activates iron regulatory factor in macrophages. *EMBO J* 1993; **12**: 3643–3649.
32. Weiss G, Goossen B, Doppler W, *et al.* Translational regulation via iron-responsive elements by the nitric oxide/NO-synthase pathway. *EMBO J* 1993; **12**: 3651–3657.
33. Weiss G, Werner-Felmayer G, Werner ER, Grunewald K, Wachter H, Hentze MW. Iron regulates nitric oxide synthase activity by controlling nuclear transcription. *J Exp Med* 1994; **180**: 969–976.
34. Takagi K, Nukaya I, Yasukawa K, Suketa Y. Inhibitory mechanisms of antibody production by nitrogen oxides released from activated macrophages during the immune response: relationship to energy consumption. *Immunol Cell Biol* 1994; **72**: 241–248.
35. Albina JE, Abate JA, Henry WL Jr. Nitric oxide production is required for murine resident peritoneal macrophages to suppress mitogen-stimulated T cell proliferation. Role of IFN-γ in the induction of the nitric oxide-synthesizing pathway. *J Immunol* 1991; **147**: 144–148.
36. Darley-Usmar VM, Hogg N, O'Leary WJ, Wilson MT, Moncada S. The simultaneous generation of superoxide anion and nitric oxide can initiate lipid peroxidation in human low density lipoprotein. *Free Rad Res Communs* 1992; **17**: 9–20.
37. Albina JE, Cui S, Mateo RB, Reichner JS. Nitric oxide-mediated apoptosis in murine peritoneal macrophages. *J Immunol* 1993; **150**: 5080–5085.
38. Sarih M, Souvannavong V, Adam A. Nitric oxide synthase induces macrophage death by apoptosis. *Biochem Biophys Res Commun* 1993; **191**: 503–508.
39. Albina JE, Mastrofrancesco B. Modulation of glucose metabolism in macrophages by products of nitric oxide synthase. *Am J Physiol* 1993; **264**: C1594–C1599.
40. Corraliza IM, Campo ML, Fuentes JM, Campos-Portuguez S, Soler G. Parallel induction of nitric oxide and glucose-6-phosphate dehydrogenase in activated bone marrow derived macrophages. *Biochem Biophys Res Commun* 1993; **196**: 342–347.
41. Weisz A, Oguchi S, Cicatiello L, Esumi H. Dual mechanism for the control of inducible-type NO synthase gene expression in macrophages during activation by interferon-gamma and bacterial lipopolysaccaride. Transcriptional and post-transcriptional regulation. *J Biol Chem* 1994; **269**: 8324–8333.
42. Knowles RG, Merrett M, Salter M, Moncada S. Differential induction of brain, lung and liver nitric oxide synthase by endotoxin in the rat. *Biochem J* 1990; **270**: 833–836.
43. Cunha FQ, Assreuy J, Moss DW, *et al.* Differential induction of nitric oxide synthase in various organs of the mouse during endotoxaemia: role of TNF-alpha and IL-1-beta. *Immunology* 1994; **81**: 211–215.
44. Akubue PI, Stohs SJ. Endrin-induced production of nitric oxide by rat peritoneal macrophages. *Toxicol Lett* 1992; **62**: 311–316.
45. Mulligan MS, Moncada S, Ward PA. Protective effects of inhibitors of nitric oxide synthase in immune complex-induced vasculitis. *Br J Pharmacol* 1992; **107**: 1159–1162.
46. Huot AE, Hacker MP. Role of reactive nitrogen intermediate production in alveolar macrophage-mediated cytostatic activity induced by bleomycin lung damage in rats. *Cancer Res* 1990; **50**: 7863–7866.
47. Andrade J, Conde M, Sobrino F, Bedoya FJ. Activation of peritoneal macrophages during the prediabetic phase in low-dose streptozotocin-treated mice. *FEBS Lett* 1993; **327**: 32–34.
48. Thomsen LL, Ching L-M, Zhuang L, Gavin JB, Baguley BC. Tumor-dependent increased plasma nitrate concentrations as an indication of the antitumor effect of flavone-8-acetic acid and analogues in mice. *Cancer Res* 1991; **51**: 77–81.

49. Thomsen LL, Ching L-M, Joseph WR, Baguley BC, Gavin JB. Nitric oxide production in endotoxin-resistant $C_3H$/HeJ mice stimulated with flavone-8-acetic acid and xanthenone-4-acetic acid analogues. *Biochem Pharmacol* 1992; **43**: 2401–2406.
50. Punjabi CJ, Laskin JD, Hwang SM, MacEachern L, Laskin DL. Enhanced production of nitric oxide by bone marrow cells and increased sensitivity to macrophage colony-stimulating factor (CSF) and granulocyte-macrophage CSF after benzene treatment of mice. *Blood* 1994; **83**: 3255–3263.
51. Pendino KJ, Laskin JD, Shuler RL, Punjabi CJ, Laskin DL. Enhanced production of nitric oxide by rat alveolar macrophages after inhalation of a pulmonary irritant is associated with increased expression of nitric oxide synthase. *J Immunol* 1993; **151**: 7196–7205.
52. Kubes P, Susuki M, Granger DN. Nitric oxide: an endogenous modulator of leukocyte adhesion. *Proc Natl Acad Sci USA* 1991; **88**: 4651–4655.
53. Hughes SR, Williams TJ, Brain SD. Evidence that endogenous nitric oxide modulates oedema formation induced by substance P. *Eur J Pharmacol* 1990; **191**: 481–484.
54. Witztum JL, Steinberg D. Role of oxidized low density lipoprotein in atherogenesis. *J Clin Invest* 1991; **88**: 1785–1792.
55. Jessup W, Mohr D, Gieseg SP, Dean RT, Stocker R. The participation of nitric oxide in cell free- and its restriction of macrophage-mediated oxidation of low-density lipoprotein. *Biochim Biophys Acta* 1992; **1180**: 73–82.
56. Jessup W, Dean RT. Autoinhibition of murine macrophage-mediated oxidation of low-density lipoprotein by nitric oxide synthesis. *Atherosclerosis* 1993; **101**: 145–155.
57. Yates MT, Lambert LE, Whitten JP, *et al.* A protective role for nitric oxide in the oxidative modification of low density lipoproteins by mouse macrophages. *FEBS Lett* 1992; **309**: 135–138.
58. Hogg N, Kalyanaraman B, Joseph J, Struck A, Parthasarathy S. Inhibition of low-density lipoprotein oxidation by nitric oxide. Potential role in atherogenesis. *FEBS Lett* 1993; **334**: 170–174.
59. Matthys KE, Van Hove CE, Jorens PG, *et al.* Oxidized low density lipoprotein affects prostaglandin production and nitric oxide synthase activity in macrophages. *Br J Pharmacol* 1994; (in press). [Abstract]
60. Jorens PG, Rosseneu M, Devreese A-M, Bult H, Marescau B, Herman AG. Diminished capacity to release metabolites of nitric oxide synthase in macrophages loaded with oxidized low-density lipoproteins. *Eur J Pharmacol* 1992; **212**: 113–115.
61. Yang X, Cai B, Sciacca RR, Cannon PJ. Inhibition of inducible nitric oxide synthase in macrophages by oxidized low-density lipoproteins. *Circ Res* 1994; **74**: 318–328.
62. Jorens PG, Rosseneu M, Devreeze M, Bult H, Marescau B, Herman AG. Decreased production of nitrite in macrophages loaded with oxidized low-density lipoproteins. In: Moncada S, Marletta MA, Hibbs JB Jr, Higgs EA, eds. *The Biology of Nitric Oxide.* London: Portland Press Ltd, 1992; 34–37.
63. Matthys KE, Jorens PG, Marascau B, Rosseneu M, Bult H, Herman AG. Oxidized lipoproteins suppress nitric oxide synthase in macrophages: study of glucocorticoid receptor involvement. *Med Inflammation* 1994; **3**: 323–327.
64. Bolton EJ, Jessup W, Stanley KK, Dean RT. Enhanced LDL oxidation by murine macrophage foam cells and their failure to secrete nitric oxide. *Atherosclerosis* 1994; **106**: 213–223.
65. Verbeuren TJ, Bonhomme E, Laubie M, Simonet S. Evidence for induction of non-endothelial NO-synthase in aortas of cholesterol-fed rabbits. *J Cardiovasc Pharmacol* 1993; **21**: 841–845.
66. Bosmans JM, Bult H, Vrints CJM, Kockx MM, Claeys M, Snoeck JP, Herman AG. Balloon-angioplasty leads to induction of a vascular NO-synthase. In: Moncada S, Feelisch M, Busse R, Higgs EA, eds. *The Biology of Nitric Oxide. Volume 3. Physiology and clinical aspects.* London: Portland Press Ltd, 1994; (in press).
67. Joly CA, Schini VB, Vanhoutte PM. Balloon injury and interleukin-1β induce nitric oxide synthase activity in rat carotid arteries. *Circ Res* 1992; **71**: 331–338.
68. Stuehr DJ, Marletta MA. Mammalian nitrate biosynthesis: mouse macrophages produce nitrite and nitrate in response to *Escherichia coli* lipopolysaccharide. *Proc Natl Acad Sci USA* 1985; **82**: 7738–7742.
69. Fast DJ, Shannon BJ, Herriott MJ, Kennedy MJ, Rummage JA, Leu RW. Staphylococcal exotoxins stimulate nitric oxide-dependent murine macrophage tumoricidal activity. *Infect Immunon* 1991; **59**: 2987–2993.
70. Zembowicz A, Vane JR, Induction of nitric oxide synthase activity by toxic shock syndrome toxin 1 in a macrophage-monocyte cell line. *Proc Natl Acad Sci USA* 1992; **89**: 2051–2055.
71. Cunha FQ, Moss DW, Leal LMCC, Moncada S, Liew FY. Induction of macrophage parasiticidal activity by *Staphylococcus aureus* and exotoxins through the nitric oxide synthesis pathway. *Immonology* 1993; **78**: 563–567.
72. Ruschmeyer D, Thude H, Mühlradt PF. MDHM, a macrophage-activating product of *Mycoplasma fermentans*, stimulates murine macrophages to synthesize nitric oxide and become tumoricidal. *FEMS Immunol Med Microbiol* 1993; **7**: 223–230.
73. Hauschildt S, Bassenge E, Bessler W, Busse R, Mülsch A. L-Arginine-dependent nitric oxide formation and nitrite release in bone marrow-derived macrophages stimulated with bacterial lipopeptide and lipopolysaccharide. *Immunology* 1990; **70**: 332–337.
74. Ding AH, Nathan CF, Stuehr DJ. Release of reactive nitrogen intermediates and reactive oxygen intermediates from mouse peritoneal macrophages. Comparison of activating cytokines and evidence for independent production. *J Immunol* 1988; **14**: 2407–2412.
75. Cunha FQ, Weiser WY, David JR, Moss DW, Moncada S, Liew FY. Recombinant migration inhibitory factor induces nitric oxide synthase in murine macrophages. *J Immunol* 1993; **150**: 1908–1912.
76. Buchmüller-Rouiller Y, Corradin SB, Mauël J. Macrophage activation for intracellular killing as induced by a $Ca^{2+}$ ionophore. *Biochem J* 1992; **284**: 387–392.
77. Thomsen LL, Ching L-M, Baguley BC. Evidence for the production of nitric oxide by activated macrophages treated with the antitumor agents flavone-8-acetic acid and xanthenone-4-acetic acid. *Cancer Res* 1990; **50**: 6966–6970.
78. Mina M, Kong ZL, Shinohara K, Watanabe M. Soybean trypsin inhibitor and β-amylase stimulate macrophages. *Biochem Biophys Res Commun* 1990; **173**: 296–301.
79. Jorens PG, Van Overveld FJ, Bult H, Vermeire PA, Herman AG. Soybean trypsin inhibitor and β-amylase induce alveolar macrophages to release nitrogen oxides. *Biochem Pharmacol* 1992; **44**: 387–390.
80. Thomas G, Ando T, Verma K, Kagan E. Asbestos-induced nitric oxide production: synergistic effect with interferon-gamma. *Ann NY Acad Sci* 1994; **725**: 207–212.
81. Sowa G, Przewlocki R. cAMP analogues and cholera toxin stimulate the accumulation of nitrite in rat peritoneal macrophage cultures. *Eur J Pharmacol* 1994; **266**: 125–129.
82. Hauschildt S, Bessler WG, Scheipers P. Engagement of major histocompatibility complex class II molecules leads to nitrite production in bone marrow-derived macrophages. *Eur J Immunol* 1993; **23**: 2988–2992.
83. Isobe K-I, Nakashima I. Abundant production of nitric oxide from murine macrophages by direct stimulation of tumor cells. *Biochem Biophys Res Commun* 1993; **192**: 499–504.
84. Lavnikova N, Drapier J-C, Laskin DL. A single exogenous stimulus activates resident rat macrophages for nitric oxide production and tumor cytotoxicity. *J Leukoc Biol* 1993; **54**: 322–328.
85. Jorens PG, Van Overveld FJ, Bult H, Vermeire PA, Herman AG. L-Arginine dependent production of nitrogen oxides by rat pulmonary macrophages. *Eur J Pharmacol* 1991; **200**: 205–209.
86. Oswald IP, Afroun S, Bray D, Petit J-F, Lemaire G. Low response of BALB/c macrophages to priming and activating signals. *J Leukoc Biol* 1992; **52**: 315–322.
87. Ding A, Hwang S, Schwab R. Effect of aging on murine macrophages. Diminished response to IFN-gamma for enhanced oxidative metabolism. *J Immunol* 1994; **153**: 2146–2152.
88. Pellat-Deceunynck C, Wietzerbin J, Drapier J-C. Nicotinamide inhibits nitric oxide synthase mRNA induction in activated macrophages. *Biochem J* 1994; **297**: 53–58.
89. Hauschildt S, Scheipers P, Bessler WG. Inhibitors of poly (ADP-ribose) polymerase suppress lipopolysaccharide-induced nitrite formation in macrophages. *Biochem Biophys Res Commun* 1991; **179**: 865–871.
90. Oguchi S, Weisz A, Esumi H. Enhancement of inducible-type NO synthase gene transcription by protein synthesis inhibitors. Activation of an intracellular signal transduction pathway by low concentrations of cycloheximide. *FEBS Lett* 1994; **338**: 326–330.
91. Evans T, Carpenter A, Cohen J. Inducible nitric-oxide-synthase mRNA is transiently expressed and destroyed by a cycloheximide-sensitive process. *Eur J Biochem* 1994; **219**: 563–569.
92. Zhang X, Morrison DC. Pertussis toxin-sensitive factor differentially regulates lipopolysaccharide-induced tumor necrosis factor-α and nitric oxide production in mouse peritoneal macrophages. *J Immunol* 1993; **150**: 1011–1018.
93. Zhang X, Morrison DC. Lipopolysaccharide-induced selective priming effects on tumor necrosis factor α and nitric oxide production in mouse peritoneal macrophages. *J Exp Med* 1993; **177**: 511–516.
94. Hortelano S, Genaro AM, Bosca L. Phorbol esters induce nitric oxide synthase and increase arginine influx in cultured peritoneal macrophages. *FEBS Lett* 1993; **320**: 135–139.
95. Severn A, Wakelam MJO, Liew FY. The role of protein kinase C in the induction of nitric oxide synthesis by murine macrophages. *Biochem Biophys Res Commun* 1992; **188**: 997–1002.
96. Bosca L, Laza PA. Induction of nitric oxide release by MRC OX-44 (anti-CD53) through a protein kinase C-dependent pathway in rat macrophages. *J Exp Med* 1994; **179**: 1119–1126.
97. Sands WA, Clark JS, Liew FY. The role of a phosphatidylcholine-specific phospholipase C in the production of diacylglycerol for nitric oxide synthesis in macrophages activated by IFN-gamma and LPS. *Biochem Biophys Res Commun* 1994; **199**: 461–466.
98. Dong Z, Qi X, Xie K, Fidler IJ. Protein tyrosine kinase inhibitors decrease induction of nitric oxide synthase activity in lipopolysaccharide-responsive and lipopolysaccharide-nonresponsive murine macrophages. *J Immunol* 1993; **151**: 2717–2724.
99. Novogrodsky A, Vanichkin A, Patya M, Gazit A, Osherov N, Levitzki A. Prevention of lipopolysaccharide-induced lethal toxicity by tyrosine kinase inhibitors. *Science* 1994; **264**: 1319–1322.
100. Kamijo R, Harada H, Matsuyama T, *et al.* Requirement for transcription factor IRF-1 in NO synthase induction in macrophages. *Science* 1994; **263**: 1612–1615.
101. Xie QW, Kashiwabara Y, Nathan C. Role of transcription factor NF-kappa B/Rel in induction of nitric oxide synthase. *J Biol Chem* 1994; **269**: 4705–4708.
102. Sherman MP, Aeberhard EE, Wong VZ, Griscavage JM, Ignarro LJ. Pyrrolidine dithiocarbamate inhibits induction of nitric oxide synthase activity in rat alveolar macrophages. *Biochem Biophys Res Commun* 1993; **191**: 1301–1308.
103. Drapier J-C, Wietzerbin J, Hibbs JB Jr. Interferon-γ and tumor necrosis factor induce the L-arginine-dependent cytotoxic effector mechanism in murine macrophages. *Eur J Immunol* 1988; **18**: 1587–1592.
104. Corradin SB, Heumann D, Gallay P, Smith J, Mauël J, Glauser MP. Bactericidal/permeability-increasing protein inhibits induction of macrophage nitric oxide production by lipopolysaccharide. *J Infect Dis* 1994; **169**: 105–111.
105. Corradin SB, Mauël J, Gallay P, Heumann D, Ulevitch RJ, Tobias PS. Enhancement of murine macrophage binding of and response to bacterial lipopolysaccharide (LPS) by LPS-binding protein. *J Leukoc Biol* 1992; **52**: 363–368.
106. Lorsbach RB, Murphy WJ, Lowenstein CJ, Snyder SH, Russell SW. Expression of the nitric oxide synthase gene in mouse macrophages activated for tumor cell killing. *J Biol Chem* 1993; **268**: 1908–1913.

107. Green SJ, Chen T-Y, Crawford RM, Nacy CA, Morrison DC, Meltzer MS. Cytotoxic activity and production of toxic nitrogen oxides by macrophages treated with IFN-γ and monoclonal antibodies against the 73-kDa lipopolysaccharide receptor. *J Immunol* 1992; **149**: 2069–2075.
108. Amber IJ, Hibbs JB Jr, Parker CJ, Johnson BB, Taintor RR, Vavrin Z. Activated macrophage conditioned medium: identification of the soluble factors inducing cytotoxicity and the L-arginine dependent effector mechanism. *J Leukoc Biol* 1991; **49**: 610–620.
109. Bogdan C, Vodovotz Y, Paik J, Xie Q-W, Nathan C. Traces of bacterial lipopolysaccharide suppress IFN-γ-induced nitric oxide synthase gene expression in primary mouse macrophages. *J Immunol* 1993; **151**: 301–309.
110. Severn A, Xu D, Doyle J, *et al.* Pre-exposure of murine macrophages to lipopolysaccharide inhibits the induction of nitric oxide synthase and reduces leishmanicidal activity. *Eur J Immunol* 1993; **23**: 1711–1714.
111. Corradin SB, Buchmüller-Rouiller Y, Mauël J. Phagocytosis enhances murine macrophage activation by interferon-γ and tumor necrosis factor-α. *Eur J Immunol* 1991; **21**: 2553–2558.
112. Cunha FQ, Assreuy J, Moncada S, Liew FY. Phagocytosis and induction of nitric oxide synthase in murine macrophages. *Immunology* 1993; **79**: 408–411.
113. Kremsner PG, Nüssler A, Neifer S, *et al.* Malaria antigen and cytokine-induced production of reactive nitrogen intermediates by murine macrophages: no relevance to the development of experimental cerebral malaria. *Immunology* 1993; **78**: 286–290.
114. Green SJ, Crawford RM, Hockmeyer JT, Meltzer MS, Nacy CA. Leishmania major amastigotes initiate the L-arginine-dependent killing mechanism in IFN-γ-stimulated macrophages by induction of tumor necrosis factor-α. *J Immunol* 1990; **145**: 4290–4297.
115. Green SJ, Mellouk S, Hoffman SL, Meltzer MS, Nacy CA. Cellular mechanisms of nonspecific immunity to intracellular infection: cytokine-induced synthesis of toxic nitrogen oxides from L-arginine by macrophages and hepatocytes. *Immunol Lett* 1990; **25**: 15–20.
116. Langermans JAM, Van Der Hulst MEB, Nibbering PH, Hiemstra PS, Fransen L, Van Furth R. IFN-γ-induced L-arginine-dependent toxoplasmastatic activity in murine peritoneal macrophages is mediated by endogenous tumor necrosis factor-α. *J Immunol* 1992; **148**: 568–574.
117. Liew FY, Li Y, Millott S. Tumour necrosis factor (TNF-alpha) in Leishmaniasis. II. TNF-alpha-induced macrophage leishmanicidal activity is mediated by nitric oxide from L-arginine. *Immunology* 1990; **71**: 556–559.
118. Liew FY, Li Y, Millott S. Tumor necrosis factor-alpha synergizes with IFN-γ in mediating killing of Leishmania major through the induction of nitric oxide. *J Immunol* 1990; **145**: 4306–4310.
119. Munoz-Fernandez MA, Fernandez MA, Fresno M. Synergism between tumor necrosis factor-α and interferon-γ on macrophage activation for the killing of intracellular *Trypsanosoma cruzi* through a nitric oxide-dependent mechanism. *Eur J Immunol* 1992; **22**: 301–307.
120. Leu RW, Stewart CA, Herriott MJ, Fast DJ, Rummage JA. Inhibitor of C1q secretion suppresses the macrophage response to lipid A for nitric oxide but not for TNF production: evidence for a role of C1q in autocrine binding of TNF. *Immunobiol* 1993; **188**: 242–258.
121. Higuchi M, Higashi N, Taki H, Osawa T. Cytolytic mechanisms of activated macrophages. Tumor necrosis factor and L-arginine-dependent mechanisms act synergistically as the major cytolytic mechanisms of activated macrophages. *J Immunol* 1990; **144**: 1425–1431.
122. Birkland TP, Sypek JP, Wyler DJ. Soluble TNF and membrane TNF expressed on CD4+ T lymphocytes differ in their ability to activate macrophage antileishmanial defense. *J Leukoc Biol* 1992; **51**: 296–299.
123. Jorens PG, Van Overveld FJ, Bult H, Vermeire PA, Herman AG. Muramylpeptide and granulocyte macrophage-colony stimulating factor enhance interferon-γ induced nitric oxide production by rat alveolar macrophages. *Agents Actions* 1993; **38**: 100–105.
124. Zhang X, Alley EW, Russell SW, Morrison DC. Necessity and sufficiency of beta interferon for nitric oxide production in mouse peritoneal macrophages. *Infect Immun* 1994; **62**: 33–40.
125. Rutherford MS, Schook LB. Macrophage function in response to PGE12, L-arginine deprivation, and activation by colony-stimulating factors is dependent on hematopoietic stimulus. *J Leukoc Biol* 1992; **52**: 228–235.
126. Rutherford MS, Schook LB. Differential immunocompetence of macrophages derived using macrophage or granulocyte-macrophage colony-stimulating factor. *J Leukoc Biol* 1992; **51**: 69–76.
127. Sodhi A, Suresh A. Production of reactive nitrogen intermediates by bone marrow-derived macrophages on treatment with cisplatin *in vitro*. *Clin Exp Immunol* 1992; **89**: 502–508.
128. Deng W, Thiel B, Tannenbaum CS, Hamilton TA, Stuehr DJ. Synergistic cooperation between T cell lymphokines for induction of the nitric oxide synthase gene in murine peritoneal macrophages. *J Immunol* 1993; **151**: 322–329.
129. Cox GW, Melillo G, Chattopadhyay U, Mullet D, Fertel RH, Varesio L. Tumor necrosis factor-α-dependent production of reactive nitrogen intermediates mediates IFN-γ plus IL-2-induced murine macrophage tumoricidal activity. *J Immunol* 1992; **149**: 3290–3296.
130. Jiang H, Stewart CA, Fast DJ, Leu RW. Tumor target-derived soluble factor synergizes with IFN-γ and IL-2 to activate macrophages for tumor necrosis factor and nitric oxide production to mediate cytotoxicity of the same target. *J Immunol* 1992; **149**: 2137–2146.
131. Herriott MJ, Jiang H, Stewart CA, Fast DJ, Leu RW. Mechanistic differences between migration inhibitory factor (MIF) and IFN-gamma for macrophage activation. MIF and interferon-gamma synergize with lipid A to mediate migration inhibition, but only interferon-gamma induces production of TNF-α and NO. *J Immunol* 1993; **150**: 4524–4531.
132. Ding A, Nathan CF, Graycar J, Derynck R, Stuehr DJ, Srimal S. Macrophage deactivating factor and transforming growth factors-beta 1 -beta 2 and -beta 3 inhibit induction of macrophage nitrogen oxide synthesis by IFN-γ. *J Immunol* 1990; **145**: 940–944.
133. Gazzinelli RT, Oswald IP, Hieny S, James LS, Sher A. The microbicidal activity of interferon-γ-treated macrophages against *Trypsanosoma cruzi* involves an L-arginine-dependent, nitrogen oxide-mediated mechanism inhibitable by interleukin-10 and transforming growth factor-β. *Eur J Immunol* 1992; **22**: 2501–2506.
134. Al-Ramadi BK, Meissler JJ Jr, Huang D, Eisenstein TK. Immunosuppression induced by nitric oxide and its inhibition by interleukin-4. *Eur J Immunol* 1992; **22**: 2249–2254.
135. Cunha FQ, Moncada S, Liew FY. Interleukin-10 (IL-10) inhibits the induction of nitric oxide synthase by interferon-γ in murine macrophages. *Biochem Biophys Res Commun* 1992; **182**: 1155–1159.
136. Doyle AG, Herbein G, Montaner LJ, *et al.* Interleukin-13 alters the activation state of murine macrophages *in vitro*: comparison with interleukin-4 and interferon-gamma. *Eur J Immunol* 1994; **24**: 1441–1445.
137. Doherty TM, Kastelein R, Menon S, Andrade S, Coffman RL. Modulation of murine macrophage function by IL-13. *J Immunol* 1993; **151**: 7151–7160.
138. Liew FY, Li Y, Severn A, *et al.* A possible novel pathway of regulation by murine T helper type 2 ($T_h2$) cells of a $T_h1$ cell activity via the modulation of the induction of nitric oxide synthase on macrophages. *Eur J Immunol* 1991; **21**: 2489–2494.
139. Bogdan C, Stenger S, Röllinghoff M, Solbach W. Cytokine interactions in experimental cutaneous leishmaniasis. Interleukin 4 synergizes with interferon-γ to activate murine macrophages for killing of Leishmania major amastigotes. *Eur J Immunol* 1991; **21**: 327–333.
140. Oswald IP, Wynn TA, Sher A, James SL. Interleukin 10 inhibits macrophage microbicidal activity by blocking the endogenous production of tumor necrosis factor α required as a costimulatory factor for interferon γ-induced activation. *Proc Natl Acad Sci USA* 1992; **89**: 8676–8680.
141. Corradin SB, Fasel N, Buchmüller-Rouiller Y, Ransijn A, Smith J, Mauël J. Induction of macrophage nitric oxide production by interferon-γ and tumor necrosis factor-α is enhanced by interleukin-10. *Eur J Immunol* 1993; **23**: 2045–2048.
142. Cenci E, Romani L, Mencacci A, *et al.* Interleukin-4 and interleukin-10 inhibit nitric oxide-dependent macrophage killing of *Candida albicans*. *Eur J Immunol* 1993; **23**: 1034–1038.
143. Oswald IP, Gazzinelli RT, Sher A, James SL. IL-10 synergizes with IL-4 and transforming growth factor-β to inhibit macrophage cytotoxic activity. *J Immunol* 1992; **148**: 3578–3582.
144. Nelson BJ, Ralph P, Green SJ, Nacy CA. Differential susceptibility of activated macrophage cytotoxic effector reactions to the suppressive effects of transforming growth factor-β1. *J Immunol* 1991; **146**: 1849–1857.
145. Förstermann U, Schmidt HHHW, Kohlhaas KL, Murad F. Induced RAW 264.7 macrophages express soluble and particulate nitric oxide synthase: inhibition by transforming growth factor-β. *Eur J Pharmacol (Mol Pharmacol)* 1992; **225**: 161–165.
146. Gilbert RS, Herschman HR. Transforming growth factor beta differentially modulates the inducible nitric oxide synthase gene in distinct cell types. *Biochem Biophys Res Commun* 1993; **195**: 380–384.
147. Vodovotz Y, Bogdan C, Paik J, Xie Q-W, Nathan C. Mechanisms of suppression of macrophage nitric oxide release by transforming growth factors β. *J Exp Med* 1993; **178**: 605–613.
148. Rojas A, Delgado R, Glaria L, Palacios M. Monocyte chemotactic protein-1 inhibits the induction of nitric oxide synthase in J774 cells. *Biochem Biophys Res Commun* 1993; **196**: 274–279.
149. Bernard C, Merval R, Esposito B, Tedgui A. Elevated temperature accelerates and amplifies the induction of nitric oxide synthesis in rat macrophages. *Eur J Pharmacol* 1994; **270**: 115–118.
150. Melillo G, Cox GW, Radzioch D, Varesio L. Picolinic acid, a catabolite of L-tryptophan, is a costimulus for the induction of reactive nitrogen intermediate production in murine macrophages. *J Immunol* 1993; **150**: 4031–4040.
151. Tonetti M, Sturla L, Bistolfi T, Benatti U, De Flora A. Extracellular ATP potentiates nitric oxide synthase expression induced by lipopolysaccharide in RAW 264.7 murine macrophages. *Biochem Biophys Res Commun* 1994; **203**: 430–435.
152. Foresta P, Ruggiero V, Albertoni C, Pacello L, Leoni B, Martelli EA. *In vitro* activation of murine peritoneal exudate cells (PEC) and peritoneal macrophages by ST 789. *Int J Immunopharmac* 1992; **14**: 1061–1068.
153. Barratt GM, Raddassi K, Petit J-F, Tenu J-P. MDP and LPS act synergistically to induce arginine-dependent cytostatic activity in rat alveolar macrophages. *Int J Immunopharmac* 1991; **13**: 159–165.
154. Kohama Y, Iida K, Tanaka K, *et al.* Studies on thermophile products. VI. Activation of mouse peritoneal macrophages by bis(2-hydroxyethyl) trisulfide. *Biol Pharm Bull* 1993; **16**: 973–977.
155. Cillari E, Arcoleo F, Dieli M, *et al.* The macrophage-activating tetrapeptide tuftsin induces nitric oxide synthesis and stimulates murine macrophages to kill Leishmania parasites *in vitro*. *Infect Immun* 1994; **62**: 2649–2652.
156. Goodrum KJ, McCormick LL, Schneider B. Group B streptococcus-induced nitric oxide production in murine macrophages is CR3 (CD11b/DC18) dependent. *Infect Immun* 1994; **62**: 3102-3107.
157. Buchmüller-Rouiller Y, Corradin SB, Smith J, Mauël J. Effect of increasing intravesicular pH on nitrite production and leishmanicidal activity of activated macrophages. *Biochem J* 1994; **301**: 243–247.

158. Manthey CL, Perera P-Y, Salkowski CA, Vogel SN. Taxol provides a second signal for murine macrophage tumoricidal activity. *J Immunol* 1994; **152**: 825–831.
159. Marczin N, Papapetropoulos A, Jilling T, Catravas JD. Prevention of nitric oxide synthase induction in vascular smooth muscle cells by microtubule deploymerizing agents. *Br J Pharmacol* 1993; **109**: 603–605.
160. Szabo C, Wu C-C, Mitchell JA, Gross SS, Thiemermann C, Vane JR. Platelet-activating factor contributes to the induction of nitric oxide synthase by bacterial lipopolysaccharide. *Circ Res* 1993; **73**: 991–999.
161. Renier G, Skamene E, DeSanctis J, Radzioch D. Dietary n-3 polyunsaturated fatty acids prevent the development of atherosclerotic lesions in mice. Modulation of macrophage secretory activities. *Arterioscler Thromb* 1993; **13**: 1515–1524.
162. Chaet MS, Garcia VF, Arya G, Ziegler MM. Dietary fish oil enhances macrophage production of nitric oxide. *J Surg Res* 1994; **57**: 65–68.
163. Imai Y, Kolb H, Burkart V. Nitric oxide production from macrophages is regulated by arachidonic acid metabolites. *Biochem Biophys Res Commun* 1993; **197**: 105–109.
164. Ryoyama K, Nomura T, Nakamura S. Inhibition of macrophage nitric oxide production by arachidonate-cascade inhibitors. *Cancer Immunol Immunother* 1993; **37**: 385–391.
165. Gaillard T, Mülsch A, Busse R, Klein H, Decker K. Regulation of nitric oxide production by stimulated rat Kupffer cells. *Pathobiology* 1991; **59**: 280–283.
166. Koide M, Kawahara Y, Nakayama I, Tsuda T, Yokoyama M. Cyclic AMP-elevating agents induce an inducible type of nitric oxide synthase in cultured vascular smooth muscle cells. *J Biol Chem* 1993; **268**: 24959–24966.
167. Imai T, Hirata Y, Kanno K, Marumo F. Induction of nitric oxide synthase by cyclic AMP in rat vascular smooth muscle cells. *J Clin Invest* 1994; **93**: 543–549.
168. Marotta P, Sautebin L, Di Rosa M. Modulation of the induction of nitric oxide synthase by eicosanoids in the murine macrophage cell line J774. *Br J Pharmacol* 1992; **107**: 640–641.
169. Raddassi K, Petit J-F, Lemaire G. LPS-induced activation of primed murine peritoneal macrophages is modulated by prostaglandins and cyclic nucleotides. *Cell Immunol* 1993; **149**: 50–64.
170. Buchmüller-Rouiller Y, Corradin SB, Mauël J. Differential effects of prostaglandins on macrophage activation induced by calcium ionophore A23187 or IFN-γ. *J Immunol* 1992; **148**: 1171–1175.
171. Bulut V, Severn A, Liew FY. Nitric oxide production by murine macrophages is inhibited by prolonged elevation of cyclic AMP. *Biochem Biophys Res Commun* 1993; **195**: 1134–1138.
172. Feinstein DL, Galea E, Reis DJ. Norepinephrine suppresses inducible nitric oxide synthase activity in rat astroglial cultures. *J Neurochem* 1993; **60**: 1945–1948.
173. Salvemini D, Misko TP, Masferrer JL, Seibert K, Currie MG, Needleman P. Nitric oxide activates cyclooxygenase enzymes. *Proc Natl Acad Sci USA* 1993; **90**: 7240–7244.
174. Griscavage JM, Rogers NE, Sherman MP, Ignarro LJ. Inducible nitric oxide synthase from a rat alveolar macrophage cell line is inhibited by nitric oxide. *J Immunol* 1993; **151**: 6329–6337.
175. Assreuy J, Cunha FQ, Liew FY, Moncada S. Feedback inhibition of nitric oxide synthase activity by nitric oxide. *Br J Pharmacol* 1993; **108**: 833–837.
176. Hattori R, Kosuga K, Eizawa H, *et al.* Stabilization of inducible nitric oxide synthase by monoclonal antibodies. *Hybridoma* 1993; **12**: 763–770.
177. Vodovotz Y, Kwon NS, Pospischil M, Manning J, Paik J, Nathan C. Inactivation of nitric oxide synthase after prolonged incubation of mouse macrophages with IFN-γ and bacterial lipopolysaccharide. *J Immunol* 1994; **152**: 4110–4118.
178. Barbul A. Physiology and pharmacology of arginine. In: Moncada S, Higgs EA, eds. *Nitric Oxide from L-Arginine: a bioregulatory system.* Amsterdam: Elsevier Science Publ. B.V., 1990; 317–329.
179. Albina JE, Mills CD, Henry WL Jr, Caldwell MD. Temporal expression of different pathways of L-arginine metabolism in healing wounds. *J Immunol* 1990; **144**: 3877–3880.
180. Benninghoff B, Lehmann V, Eck H-P, Dröge W. Production of citrulline and ornithine by interferon-γ treated macrophages. *Int Immunol* 1991; **3**: 413–417.
181. Daghigh F, Fukuto JM, Ash DE. Inhibition of rat liver arginase by an intermediate in NO biosynthesis, NG-hydroxy-L-arginine: implications for the regulation of nitric oxide biosynthesis by arginase. *Biochem Biophys Res Commun* 1994; **202**: 174–180.
182. Nussler AK, Billiar TR, Liu Z-Z, Morris SM Jr. Coinduction of nitric oxide synthase and argininosuccinate synthetase in a murine macrophage cell line. *J Biol Chem* 1994; **269**: 1257–1261.
183. Wu G, Brosnan JT. Macrophages can convert citrulline into arginine. *Biochem J* 1992; **281**: 45–48.
184. Baydoun AR, Bogle RG, Pearson JD, Mann GE. Selective inhibition by dexamethasone of induction of NO synthase, but not of induction of L-arginine transport, in activated murine macrophage J774 cells. *Br J Pharmacol* 1993; **110**: 1401–1406.
185. Bogle RG, Baydoun AR, Pearson JD, Moncada S, Mann GE. L-Arginine transport is increased in macrophages generating nitric oxide. *Biochem J* 1992; **284**: 15–18.
186. Sato H, Fujiwara M, Bannai S. Effect of lipopolysaccharide on transport and metabolism of arginine in mouse peritoneal macrophages. *J Leukoc Biol* 1992; **52**: 161–164.
187. Kwon NS, Nathan CF, Stuehr DJ. Reduced biopterin as a cofactor in the generation of nitrogen oxides by murine macrophages. *J Biol Chem* 1989; **264**: 20496–20501.
188. Tayeh MA, Marletta MA. Macrophage oxidation of L-arginine to nitric oxide, nitrite, and nitrate. Tetrahydrobiopterin is required as a cofactor. *J Biol Chem* 1989; **264**: 19654–19658.
189. Schoedon G, Schneemann M, Hofer S, Guerrero L, Blau N, Schaffner A. Regulation of the L-arginine-dependent and tetrahydrobiopterin-dependent biosynthesis of nitric oxide in murine macrophages. *Eur J Biochem* 1993; **213**: 833–839.
190. Werner ER, Werner-Felmayer G, Fuchs D, *et al.* Tetrahydrobiopterin biosynthetic activities in human macrophages, fibroblasts, THP-1, and T 24 cells. *J Biol Chem* 1991; **265**: 3189–3192.
191. Werner-Felmayer G, Werner ER, Fuchs D, Hausen A, Reibnegger G, Wachter H. Tetrahydrobiopterin-dependent formation of nitrite and nitrate in murine fibroblasts. *J Exp Med* 1990; **172**: 1599–1607.
192. Meyer KC, Cornwell R, Carlin JM, *et al.* Effects of interferons β or gamma on neopterin biosynthesis and tryptophan degradation by human alveolar macrophages *in vitro*: synergy with lipopolysaccharide. *Am J Respir Cell Mol Med* 1992; **6**: 639–646.
193. Jorens PG, Van Overveld FJ, Bult H, Vermeire PA, Herman AG. Pterins inhibit nitric oxide synthase activity in rat alveolar macrophages. *Br J Pharmacol* 1992; **107**: 1088–1091.
194. Nichol CA, Smith GK, Duch DS. Biosynthesis and metabolism of tetrahydrobiopterin and molybdopterin. *Ann Rev Biochem* 1995; **54**: 729–764.
195. Stuehr DJ, Fasehun OA, Soo Kwon N, *et al.* Inhibition of macrophage and endothelial cell nitric oxide synthase by diphenyleneiodonium and its analogs. *FASEB J* 1991; **5**: 98–103.
196. Kilbourn R, Lopez-Berestein G. Protease inhibitors block the macrophage-mediated inhibition of tumor cell mitochondrial respiration. *J Immunol* 1990; **144**: 1042–1045.
197. Jorens PG, Van Overveld FJ, Bult H, Vermeire PA, Herman AG. Serine-protease inhibitors modulate nitric oxide-synthase activity of alveolar macrophages. *Agents Actions* 1992; **36**: 243–247.
198. Sibille Y, Merrill WW, Cooper JA Jr, Polonski L, Gee JBG. Effects of a series of chloromethylketone protease inhibitors on superoxide release and the glutathione system in human polymorphonuclear leukocytes and alveolar macrophages. *Am Rev Respir Dis* 1984; **130**: 110–114.
199. Di Rosa M, Radomski M, Carnuccio R, Moncada S. Glucocorticoids inhibit the induction of nitric oxide synthase in macrophages. *Biochem Biophys Res Commun* 1990; **172**: 1246–1252.
200. Moncada S, Palmer RMJ. Inhibition of the induction of nitric oxide synthase by glucocorticoids: yet another explanation for their anti-inflammatory effects. *Trends Pharmacol Sci* 1991; **12**: 130–131.
201. Jorens PG, Van Overveld FJ, Bult H, Vermeire PA, Herman AG. Corticosteroids prevent the induction of nitric oxide-synthase activity in different pulmonary cells. In: De Deyn PD, Marescau B, Stalon V, Qureshi IA, eds. *Guanidino Compounds in Biology and Medicine.* London: John Libbey & Company, 1992; 97–101.
202. Shepherd VL, Cowan HB, Abdolrasulnia R, Vick S. Dexamethasone blocks the interferon-gamma-mediated downregulation of the macrophage mannose receptor. *Arch Biochem Biophys* 1994; **312**: 367–374.
203. Dileepan KN, Lorsbach RB, Stechschulte DJ. Mast cell granules inhibit macrophage-mediated lysis of mastocytoma cells (P815) and nitric oxide production. *J Leukoc Biol* 1993; **53**: 446–453.
204. Zingarelli B, Carnuccio R, Di Rosa M. Cloricromene inhibits the induction of nitric oxide synthase. *Eur J Pharmacol* 1993; **243**: 107–111.
205. Kondo Y, Takano F, Hojo H. Inhibitory effect of bisbenzylisoquinoline alkaloids on nitric oxide production in activated macrophages. *Biochem Pharmacol* 1993; **46**: 1887–1892.
206. Mehta K, McQueen T, Tucker S, Pandita R, Aggarwal BB. Inhibition by all-trans-retinoic acid of tumor necrosis factor and nitric oxide production by peritoneal macrophages. *J Leukoc Biol* 1994; **55**: 336–342.
207. Szabo C, Thiemermann C, Vane JR. Dihydropyridine antagonists and agonists of calcium channels inhibit the induction of nitric oxide synthase by endotoxin in cultured macrophages. *Biochem Biophys Res Commun* 1993; **196**: 825–830.
208. Szabo C, Mitchell JA, Gross SS, Thiemermann C, Vane JR. Nifedipine inhibits the induction of nitric oxide synthase by bacterial lipopolysaccharide. *J Pharmacol Exp Ther* 1993; **265**: 674–680.
209. Hibbs JB Jr, Taintor RR, Vavrin Z. Macrophage cytotoxicity: role for L-arginine deiminase and imino nitrogen oxidation to nitrite. *Science* 1987; **235**: 473–476.
210. McCall TB, Feelisch M, Palmer RMJ, Moncada S. Identification of *N*-iminoethyl-L-ornithine as an irreversible inhibitor of nitric oxide synthase in phagocytic cells. *Br J Pharmacol* 1991; **102**: 234–238.
211. Gross SS, Stuehr DJ, Aisaka K, Jaffe EA, Levi R, Griffith OW. Macrophage and endothelial cell nitric oxide synthesis: cell-type selective inhibition by $N^G$-aminoarginine, $N^G$-nitroarginine and $N^G$-methylarginine. *Biochem Biophys Res Commun* 1990; **170**: 96–103.
212. Lambert LE, Whitten JP, Baron BM, Cheng HC, Doherty NS, McDonald IA. Nitric oxide synthesis in the CNS endothelium and macrophages differs in its sensitivity to inhibition by arginine analogues. *Life Sci* 1991; **48**: 69–75.
213. Lambert LE, French JF, Whitten JP, Baron BM, McDonald IA. Characterization of cell selectivity of two novel inhibitors of nitric oxide synthesis. *Eur J Pharmacol* 1992; **216**: 131–134.
214. Furfine ES, Harmon MF, Paith JE, Garvey EP. Selective inhibition of constitutive nitric oxide synthase by L-$N^G$-nitroarginine. *Biochemistry* 1993; **32**: 8512–8517.
215. Feldman PL, Griffith OW, Hong H, Stuehr DJ. Irreversible inactivation of macrophage and brain nitric oxide synthase by L-$N^G$-methylarginine requires NADPH-dependent hydroxylation. *J Med* 1993; **36**: 491–496.
216. Hecker M, Mitchell JA, Harris HJ, Katsura M, Thiemermann C, Vane JR. Endothelial cells metabolize $N^G$-monomethyl-L-arginine to L-citrulline and subsequently to L-arginine. *Biochem Biophys Res Commun* 1990; **167**: 1037–1043.
217. Schmidt K, Klatt P, Mayer B. Uptake of nitric oxide synthase inhibitors by macrophage RAW 264.7. *Biochem J* 1994; **301**: 313–316.
218. Baydoun AR, Mann GE. Selective targeting of nitric oxide synthase inhibitors to system $y^+$ in activated macrophages. *Biochem Biophys Res Commun* 1994; **200**: 726–731.

219. Bogle RG, Moncada S, Pearson JD, Mann GE. Identification of inhibitors of nitric oxide synthase that do not interact with the endothelial L-arginine transporter. *Br J Pharmacol* 1992; **105**: 768–770.
220. Vallance P, Leone A, Calver A, Collier J, Moncada S. Endogenous dimethylarginine as an inhibitor of nitric oxide synthase. *J Cardiovasc Pharmacol* 1992; **20**: (Suppl 12): S60–S62.
221. Hasan K, Heesen B-J, Corbett JA, *et al.* Inhibition of nitric oxide formation by guanidines. *Eur J Pharmacol* 1993; **249**: 101–106.
222. Misko TP, Moore WM, Kasten TP, *et al.* Selective inhibition of the inducible nitric oxide synthase by aminoguanidine. *Eur J Pharmacol* 1993; **233**: 119–125.
223. MacAllister RJ, Whitley GS, Vallance P. Effects of guanidino and uremic compounds on nitric oxide pathways. *Kidney Int* 1994; **45**: 737–742.
224. Griffiths MJD, Messent M, MacAllister RJ, Evans TW. Aminoguanidine selectively inhibits inducible nitric oxide synthase. *Br J Pharmacol* 1993; **110**: 963–968.
225. Li Y, Severn A, Rogers MV, Palmer RMJ, Moncada S, Liew S, Liew FY. Catalase inhibits nitric oxide synthesis and the killing of intracellular *Leishmania major* in murine macrophages. *Eur J Immunol* 1992; **22**: 441–446.
226. Urioste S, Hall LR, Telford SR, Titus RG. Saliva of the Lyme disease vector, *Ixodes dammini*, blocks cell activation by a nonprostaglandin E2-dependent mechanism. *J Exp Med* 1994; **180**: 1077–1085.
227. Calderon C, Huang ZH, Gage DA, Sotomayor EM, Lopez DM. Isolation of a nitric oxide inhibitor from mammary tumor cells and its characterization as phosphatidyl serine. *J Exp Med* 1994; **180**: 945–958.
228. Park E, Quinn MR, Wright CE, Schuller-Levis G. Taurine chloramine inhibits the synthesis of nitric oxide and the release of tumor necrosis factor in activated RAW 264.7 cells. *J Leukoc Biol* 1993; **54**: 119–124.
229. Buchmüller-Rouiller Y, Schneider P, Betz-Corradin S, Smith J, Mauël J. 3-Amino-1,2,4-triazole inhibits macrophage NO synthase. *Biochem Biophys Res Commun* 1992; **183**: 150–155.
230. Wolff DJ, Gribin BJ. The inhibition of the constitutive and inducible nitric oxide synthase isoforms by indazole agents. *Arch Biochem Biophys* 1994; **311**: 300–306.
231. Szabo C, Southan GJ, Wood E, Thiemermann C, Vane JR. Inhibition by spermine of the induction of nitric oxide synthase in J774.2 macrophages: requirement of a serum factor. *Br J Pharmacol* 1994; **112**: 355–356.
232. Bogle RG, Whitley GS, Soo SC, Johnstone AP, Vallance P. Effect of anti-fungal imidazoles on mRNA levels and enzyme activity of inducible nitric oxide synthase. *Br J Pharmacol* 1994; **111**: 1257–1261.
233. Houdijk AP, Adolfs MJ, Bonta IL, De Jonge HR. Atriopeptins and nitroprusside provoke opposite changes in cGMP and cAMP levels in human macrophages. *Eur J Pharmacol* 1990; **179**: 413–417.
234. Tucker SD, Sivaramakrishnan MR, Klostergaard J, Lopez-Berestein G. Independence of the pattern of early cytokine release from autoregulation by nitric oxide. *J Leukoc Biol* 1991; **50**: 509–516.
235. Marcinkiewicz J, Chain BM. Differential regulation of cytokine production by nitric oxide. *Immunology* 1993; **80**: 146–159.
236. Stadler J, Harbrecht BG, Di Silvio M, *et al.* Endogenous nitric oxide inhibits the synthesis of cyclooxygenase products and interleukin-6 by rat Kupffer cells. *J Leukoc Biol* 1993; **53**: 165–172.
237. Sicher SC, Vazquez MA, Lu CY. Inhibition of macrophage Ia expression by nitric oxide. *J Immunol* 1994; **153**: 1293–1300.
238. Thomas SR, Mohr D, Stocker R. Nitric oxide inhibits indoleamine 2,3-dioxygenase activity in interferon-gamma primed mononuclear phagocytes. *J Biol Chem* 1994; **269**: 14457–14464.
239. Murray HW, Teitelbaum RF. L-Arginine-dependent reactive nitrogen intermediates and the antimicrobial effect of activated human mononuclear phagocytes. *J Infect Dis* 1992; **165**: 513–517.
240. Petit J-F, Phan-Bich L, Lemaire G, Martinache C, Lopez M. During their differentiation into macrophages, human monocytes acquire cytostatic activity independent of NO and TNFα. *Res Immunol* 1993; **144**: 277–280.
241. Cameron ML, Granger DL, Weinberg JB, Kozumbo WJ, Koren HS. Human alveolar and peritoneal macrophages mediate fungistasis independently of L-arginine oxidation to nitrite and nitrate. *Am Rev Respir Dis* 1990; **142**: 1313–1319.
242. Taylor MW, Feng G. Relationship between interferon-gamma, indolamine 2,3-dioxygenase, and tryptophan catabolism. *FASEB J* 1991; **5**: 2516–2522.
243. Padgett EL, Pruett SB. Evaluation of nitrite production by human monocyte-derived macrophages. *Biochem Biophys Res Commun* 1992; **186**: 775–781.
244. Keller R, Keist R, Joller P, Groscurth P. Mononuclear phagocytes from human bone marrow progenitor cells; morphology, surface phenotype, and functional properties of resting and activated cells. *Clin Exp Immunol* 1993; **91**: 176–182.
245. Jorens PG. Nitric oxide and interleukin-8: two mediators released by pulmonary macrophages. *PhD Thesis, UIA, University of Antwerp* 1993.
246. Fuchs D, Murr C, Reibnegger G, *et al.* Nitric oxide synthase and antimicrobial armature of human macrophages. *J Infect Dis* 1994; **169**: 224–225.
247. Denis M. Human monocytes/macrophages: NO or no NO? *J Leukoc Biol* 1994; **55**: 682–684.
248. Hibbs JB Jr, Westenfelder C, Taintor R, *et al.* Evidence for cytokine-inducible nitric oxide synthesis from L-arginine in patients receiving interleukin-2 therapy. *J Clin Invest* 1992; **89**: 867–877.
249. Ochoa JB, Udekruv AO, Billiar TR, *et al.* Nitrogen oxide levels in patients after trauma and during sepsis. *Ann Surg* 1991; **214**: 621–626.
250. Nussler AK, Di Silvio M, Billiar TR, *et al.* Stimulation of the nitric oxide synthase pathway in human hepatocytes by cytokines and endotoxin. *J Exp Med* 1992; **176**: 261–266.
251. Lelchuck R, Radomski MW, Martin JF, Moncada S. Constitutive and inducible nitric oxide synthases in human megakaryoblastic cells. *J Pharmacol Exp Ther* 1992; **262**: 1220–1224.
252. Kolios G, Robson R, Brown Z, Robertson D, Westwick J. Modulation of the inducible nitric oxide synthetase (iNOS) in the human colonic epithelial cell line HT-29. *Br J Pharmacol* 1995; (in press). (Abstract)
253. Salvemini D, De Nucci G, Gryglewski RJ, Vane JR. Human neutrophils and mononuclear cells inhibit platelet aggregation by releasing a nitric oxide-like factor. *Proc Natl Acad Sci USA* 1989; **86**: 6328–6332.
254. Denis M. Tumor necrosis factor and granulocyte macrophage-colony stimulating factor stimulate human macrophages to restrict growth of virulent *Mycobacterium avium* and to kill a virulent *M. avium*: killing effector mechanism depends on the generation of reactive nitrogen intermediates. *J Leukoc Biol* 1991; **49**: 380–387.
255. Dumarey CH, Labrousse V, Rastogi N, Vargaftig BB, Bachelet M. Selective *Mycobacterium avium*-induced production of nitric oxide by human monocyte-derived macrophages. *J Leukoc Biol* 1994; **56**: 36–40.
256. Zembala M, Siedlar M, Marcinkiewicz J, Pryjma J. Human monocytes are stimulated for nitric oxide release *in vitro* by some tumor cells but not by cytokines and lipopolysaccharide. *Eur J Immunol* 1994; **24**: 435–439.
257. Kobzik L, Bredt DS, Lowenstein CJ, *et al.* Nitric oxide synthase in human and rat lung: immunocytochemical and histochemical localization. *Am J Resp Cell & Mol Biol* 1993; **9**: 371–377.
258. Tracey WR, Xue C, Klinghofer V, *et al.* Immunochemical detection of inducible NO synthase in human lung. *Am J Physiol* 1994; **266**: L722–L727.
259. Pietraforte D, Tritarelli E, Testa U, Minetti M. gp120 HIV envelope glycoprotein increases the production of nitric oxide in human monocyte-derived macrophages. *J Leukoc Biol* 1994; **55**: 175–182.
260. Sherman MP, Loro ML, Wong VZ, Tashkin DP. Cytokine- and *Pneumocystis Carinii*-induced L-arginine oxidation by murine and human pulmonary alveolar macrophages. *J Protozool* 1991; **38**: 234S–236S.
261. Reiling N, Ulmer AJ, Duchrow M, Ernst M, Flad HD, Hauschildt S. Nitric oxide synthase: mRNA expression of different isoforms in human monocytes/macrophages. *Eur J Immunol* 1994; **24**: 1941–1944.
262. Chesrown SE, Monnier J, Visner G, Nick HS. Regulation of inducible nitric oxide synthase mRNA levels by LPS, INF-gamma, TGF-beta, and IL-10 in murine macrophage cell lines and rat peritoneal macrophages. *Biochem Biophys Res Commun.* 1994; **200**: 126–134.

**Received 30 January 1995; accepted 31 January 1995**
